# Structural basis of cyclic oligoadenylate binding to the transcription factor Csa3 outlines cross talk between type III and type I CRISPR systems

**DOI:** 10.1016/j.jbc.2022.101591

**Published:** 2022-01-14

**Authors:** Pengjun Xia, Anirudha Dutta, Kushol Gupta, Mona Batish, Vijay Parashar

**Affiliations:** 1 Department of Biological Sciences, University of Delaware, Newark, Delaware, USA; 2 Department of Medical and Molecular Sciences, University of Delaware, Newark, Delaware, USA; 3 The Department of Biochemistry and Biophysics, Perelman School of Medicine, University of Pennsylvania, Philadelphia, Pennsylvania, USA

**Keywords:** CRISPR, transcription factor, CARF domain, cyclic oligoadenylate, small-angle X-ray scattering, analytical ultracentrifugation, cA4, cyclic tetra-adenylate, CARF, CRISPR-associated Rossmann fold, Cascade, CRISPR-associated complex for antiviral defense, cOA, cyclic oligoadenylate, Crn, CRISPR ring nuclease, Csa3, CRISPR Apern 3, Csa3b_Sis_, *Sulfolobus islandicus* Csa3b, Csa3_Sso_, *Saccharolobus solfataricus* Csa3, CV, column volume, EMSA, electromobility shift assay, MGE, mobile genetic element, MST, microscale thermophoresis, PAM, protospacer adjacent motif, RNP, ribonucleoprotein, SAXS, small-angle X-ray scattering, SV-AUC, sedimentation velocity analytical ultracentrifugation, wHTH, winged helix-turn-helix

## Abstract

RNA interference by type III CRISPR systems results in the synthesis of cyclic oligoadenylate (cOA) second messengers, which are known to bind and regulate various CARF domain–containing nuclease receptors. The CARF domain–containing Csa3 family of transcriptional factors associated with the DNA-targeting type I CRISPR systems regulate expression of various CRISPR and DNA repair genes in many prokaryotes. In this study, we extend the known receptor repertoire of cOA messengers to include transcriptional factors by demonstrating specific binding of cyclic tetra-adenylate (cA4) to *Saccharolobus solfataricus* Csa3 (Csa3_Sso_). Our 2.0-Å resolution X-ray crystal structure of cA4-bound full-length Csa3_Sso_ reveals the binding of its CARF domain to an elongated conformation of cA4. Using cA4 binding affinity analyses of Csa3_Sso_ mutants targeting the observed Csa3_Sso_•cA4 structural interface, we identified a Csa3-specific cA4 binding motif distinct from a more widely conserved cOA-binding CARF motif. Using a rational surface engineering approach, we increased the cA4 binding affinity of Csa3_Sso_ up to ∼145-fold over the wildtype, which has potential applications for future second messenger-driven CRISPR gene expression and editing systems. Our in-solution Csa3_Sso_ structural analysis identified cA4-induced allosteric and asymmetric conformational rearrangement of its C-terminal winged helix-turn-helix effector domains, which could potentially be incompatible to DNA binding. However, specific *in vitro* binding of the purified Csa3_Sso_ to its putative promoter (P_Cas4a_) was found to be cA4 independent, suggesting a complex mode of Csa3_Sso_ regulation. Overall, our results support cA4-and Csa3-mediated cross talk between type III and type I CRISPR systems.

Clustered regularly interspaced short palindromic repeat (CRISPR)-Cas systems mediate prokaryotic immune response to mobile genetic elements (MGEs) (1) and have been identified in ∼40% bacterial and ∼90% archaeal genomes (2, 3, 4). These systems typically contain (i) CRISPR arrays containing alternating identical repeats and unique spacers derived from the MGEs (5, 6) and (ii) *cas* gene cassettes adjacent to the CRISPR arrays that encode helicases, nucleases, and structural proteins (7).

The hallmark property of the CRISPR-Cas systems is their ability to adapt, process, and interfere with foreign genetic material. This is accomplished through a three-stage process involving (i) spacer acquisition (adaptation), (ii) crRNA production (processing), and (iii) target interference (8). The adaptation stage involves *de novo* spacer acquisition by a complex formed by two highly conserved nucleases (Cas1 and Cas2) (9, 10). A Cas4 endonuclease further recognizes and removes a short 5′-flanking region of the prespacers called protospacer adjacent motif (PAM) ensuring integration of only mature spacers into the CRISPR array (11). During the crRNA production stage, Cas6 (or an endogenous protein) processes long CRISPR pre-RNAs into short mature crRNAs to contain a region of the extrachromosomal genetic element, a 5′-tag derived from the preceding repeat, and a 3′ end handle from the downstream repeat (12, 13, 14, 15, 16, 17). During the final stage of target interference, a ribonucleoprotein (RNP) complex, comprising the mature crRNA and Cas proteins, specifically cleaves foreign nucleic acids *via* base pairing with the crRNA (18) and by the recognition of the PAM sequence in the target (19, 20, 21, 22, 23, 24).

Based on the complexity of the associated RNP complexes, CRISPR systems are classified into two major classes (class 1 and class 2) that contain a total of six different types (types I, III, and IV for class 1 and types II, V, and VI for class 2) (25, 26). The interference RNP complexes of class 2 systems employ a single protein in complex with a crRNA, whereas class 1 systems use multisubunit RNP complexes. Owing to their simplicity and amenability to practical applications such as genome editing, the class 2 systems (containing Cas9, Cas12, and Cas13) are more well studied than class 1 systems (27, 28). The less studied class 1 systems, however, are more primitive, abundant, and widespread in prokaryotes comprising about 90% of all CRISPR-Cas systems (25, 29).

The most widespread type I and type III class 1 systems coexist in many prokaryotic genomes (30, 31, 32). Such a coexistence is well represented in genomes from a crenarchaeal order Sulfolobales, two-thirds of which harbor both the systems (4, 33, 34). For example, *Sulfolobus islandicus* encodes two CRISPR loci, one subtype I-A adaptation and two subtype III-B interference modules (34). *Saccharolobus solfataricus*, on the other hand, possesses a more extensive CRISPR system with six different CRISPR loci, which include two type I adaptation modules as well as three type I and four type III interference modules (35, 36).

The type I and III interference complexes exhibit a striking functional diversity (37). Recognition of a PAM and a crRNA complementary sequence in the target DNA by the type I interference complex (known as CRISPR-associated complex for antiviral defense or Cascade) recruits the Cas3 endonuclease for degradation of the nontarget strand (Fig. 1*B*) (38, 39). By contrast, the type III interference (cmr–csm) complexes recognize a newly transcribed phage RNA in a PAM-independent fashion by base pairing with a seed motif at the 3′ end of the crRNA (4, 30, 31, 32, 33, 34). Self-targeting during type III interference is prevented by a mismatch at the 5′ end of the crRNA (Fig. 1*A*) (40, 41). The interference by cmr–csm complexes involves degradation of the target RNA as well as the nontemplate DNA (Fig. 1*A*) (32, 42, 43, 44, 45, 46, 47). Owing to its PAM-independent functional mode, the cmr–csm complex exhibits a broad target specificity and provides a unique phage survival advantage to the prokaryotic cells coharboring the type I and III systems (48, 49).

**Figure 1:**
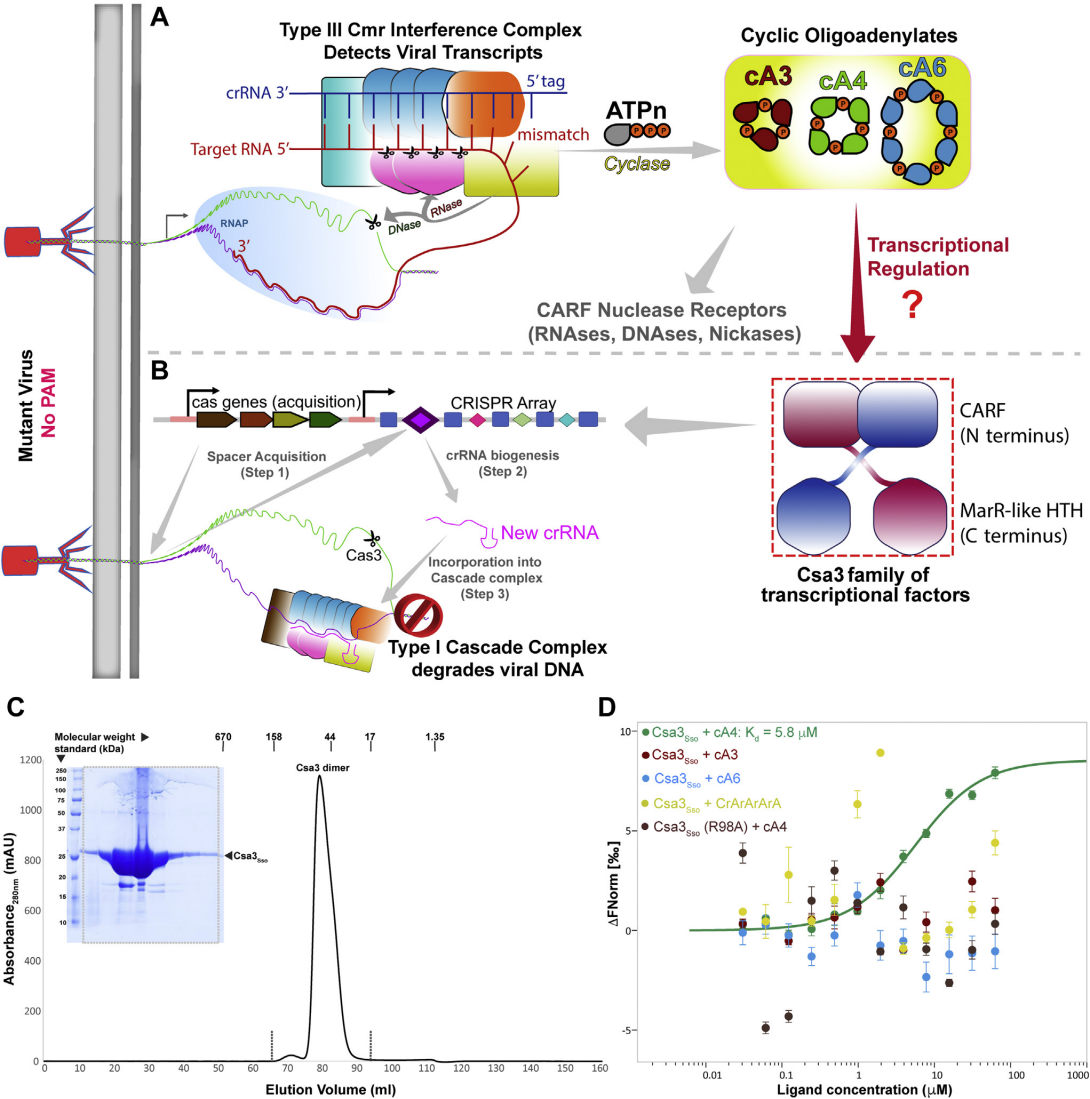
**Synthesis of cyclic oligoadenylates (cOAs) by type III interference complex, and transcriptional activation of CRISPR array and acquisition genes by Csa3a.***A*, infection by a mutant virus lacking a PAM sequence escapes DNA recognition by Cascade (type I interference complex). Type III interference complexes can use crRNAs produced from type I CRISPR loci for interference against the mutant phage. crRNA end mismatch–mediated binding of the phage transcript by type III interference complex induces ssDNA nuclease and cyclase activities of its Cas10 subunit (*yellow*) resulting in the synthesis of cA(n) (n = 3–6, with more abundant cA3, cA4, and cA6 illustrated in *yellow background*). Cas10 subunit activities are turned off by Csm3 or Cmr4 subunit (shown as *magenta ovals*)–mediated cleavage of the phage transcript. Most of the characterized cOAs receptors are nucleic acid hydrolases whose activities are regulated by cOA binding to a CARF domain. *B*, Csa3 (Csa3a-type) transcriptional factor from the type I CRISPR locus activates the transcription of acquisition *cas* genes and CRISPR arrays to facilitate acquisition of new spacers (step 1), synthesis of new crRNAs (step 2) for their incorporation into Cascade complex (step 3) for the eventual recognition and degradation of the phage DNA. Csa3a carries a CARF domain at its N terminus and is investigated as a receptor of cAn in this study. *C*, gel filtration chromatography analysis of the purified Csa3_Sso_ from *S. sulfolobus* strain P2 (UniProtKB database accession number Q97Y88, MW_theor_: 28.1 kDa) shows that Csa3_Sso_ forms dimers (MW_exper_: 58.31 kDa) in solution. *Vertical bars* above the absorbance trace indicate the peak positions of the gel filtration standards. The sodium dodecyl sulfate polyacrylamide gel electrophoresis (SDS-PAGE) picture shows the purity of Csa3_Sso_ after gel filtration. *D*, Csa3_Sso_ prefers cA4 (K_D_ of 5.8 ± 0.03 μM) over other cOAs and a linear cA4 analog. The nucleotide preference was determined by microscale thermophoresis–based binding affinity analyses.

Upon recognition of the target RNA, the palm domain of the Cas10 subunit of the cmr–csm complex synthesizes cyclic oligoadenylates (cOAs) containing 3 to 6 adenylate groups (named cA3-cA6) (Fig. 1*A*), a function that is deactivated upon target RNA cleavage (50, 51, 52). cOAs are important second messengers that orchestrate an antiviral response by primarily binding to CRISPR-associated Rossmann fold (CARF) conserved in many CRISPR-associated proteins (Fig. 1*A*) (53). cOA binding regulates the function of various nucleic acid hydrolases such as Csm6/Csx1 family ribonucleases, CRISPR ancillary nuclease 1 (Can1) proteins, and cOA-activated RNase and DNase 1 (Card1 or Can2) proteins. Furthermore, many of the CARF-containing cOA receptors harbor a cOA phosphodiesterase (also called “ring nuclease”) activity that hydrolyzes the cOA ligand into a linearized tetranucleotide (A4 > P) and then into two linear A_2_ > P species (54). The ring nuclease cOA receptors include (i) standalone host nucleases such as CRISPR ring nuclease 1 (Crn1), CRISPR ring nuclease 3 (Crn3), and CRISPR ring nuclease 2 (Crn2); (ii) stand-alone viral nuclease AcrIII1; and (iii) self-inactivating effector nuclease Csm6 (54). The ring nuclease activity of these receptors is believed to prevent a nonspecific nuclease response post MGE clearance by tightly coupling cOA-mediated response to MGE transcription (54).

Bacteriophage infections drive large global changes in the archaeal transcription. For example, infection by *S. islandicus* rod-shaped virus 2 has been shown to upregulate the expression of approximately one-third of the *S. islandicus* genome (55). Although production of cOAs by type III interference complex could conceivably mediate such transcriptional regulation, type III loci do not encode a transcription factor. The adaptation and interference cassettes of the type I systems, however, encode CRISPR Apern 3 (Csa3) family transcription factors Csa3a and Csa3b, respectively (56). The *S. islandicus* Csa3b (Csa3b_Sis_) acts as a transcriptional repressor to genes encoding subtype I-A CRISPR spacer acquisition complex, and subtype I-A target interference complex, as well as a transcriptional activator to genes encoding subtype III-B cmr interference complex (57, 58). *S. islandicus* Csa3a (Csa3a_Sis_), on the other hand, transcriptionally activates expression of CRISPR arrays, subtype I-A adaptation complex, and DNA repair proteins (59, 60). An atomic structure of the apo form of a Csa3a homolog from *S. solfataricus* (Csa3_Sso_) has been previously reported to harbor an N-terminal CARF and a C-terminal MarR-like winged helix-turn-helix (wHTH) domain (61). The Csa3_Sso_ CARF domain exhibited a dimerization-mediated 2-fold symmetric ligand-binding pocket, which was predicted to bind a four-nucleotide-long RNA (61). Consistent with this, Csa3b_Sis_ has recently been shown to bind a linear analog of cA4 (5′CAAAA3′) in a CARF domain–dependent way (57). However, despite functional significance of Csa3 transcription factors, their ligand specificity and the structural basis of ligand binding has not been reported. Here, we identify cA4 as the cognate ligand of Csa3_Sso_ and show that Csa3_Sso_ lacks ring nuclease activity *in vitro*. We determine a 2.0-Å crystal structure of Csa3_Sso_ bound to cA4 and identify Csa3_Sso_ residues important for cA4 binding. Complementary SAXS analysis indicates a cA4-induced conformational change in preformed Csa3_Sso_ dimers, suggesting that allosteric changes within the Csa3_Sso_ dimer may regulate Csa3-mediated signaling.

## Results

### Csa3_Sso_ specifically binds cyclic oligoadenylate 4

Based on previous identification of a 2-fold symmetric ligand-binding pocket at the Csa3_Sso_ (a Csa3a homolog, KEGG accession number Sso1445) dimer interface (61), and *in vitro* binding of a cA4 analog to Csa3b_Sis_ (57), we hypothesized that the Csa3 family of transcription factors from *S. solfataricus* could also be receptors of cOAs. To test specificity of binding of Csa3_Sso_ to cOA nucleotides, we purified an N-terminally His_6_-tagged fusion of Csa3_Sso_ (His_6_-Csa3_Sso_) recombinantly produced in *Escherichia coli* (Fig. 1*C*) and performed binding affinity analyses of cA3, cA4, cA6, and the linear 5′CAAAA3′ RNA analog using microscale thermophoresis (MST). Despite a nonspecific binding exhibited by high concentrations of all the cOAs (250–1000 μM) (Fig. S1), only cA4 exhibited a specific low micromolar binding to His_6_-Csa3_Sso_ with an apparent dissociation constant (K_D_) of 5.8 ± 0.03 μM (Fig. 1*D*). This is consistent with the previously observed predominance of cA4 among the cOAs produced by *S. solfataricus* Csm complex *in vitro* (50). Of note, the lack of Csa3_Sso_ binding to cA3, a second messenger also produced by CD-NTases of an alternate CBASS antiviral defense system in bacteria (62), suggests specificity of the Csa3 transcription factors to CRISPR-Cas systems. Furthermore, His_6_-Csa3_Sso_ binding to cA4 is stronger (8-fold) than the previously reported binding of Csa3b_Sis_ with the linear “cA4 analog” (5′CAAAA3′, K_D_ of 46.10 ± 8.14 μM) that was determined using surface plasmon resonance (57). However, both these Csa3 homologs from *Sulfolobus* show cA4 binding affinities lower than *Treponema succinifaciens* Card1 (K_D_ of 15 nM), which is also a cA4 receptor lacking ring nuclease activity (63). Nevertheless, considering the expected high micromolar concentrations of cA4 in the cell upon infection as discussed below (64), we believe that cA4 is the preferred ligand of Csa3_Sso_.

### The 2.0-Å crystal structure of Csa3_Sso_ bound to cA4

**Figure 2:**
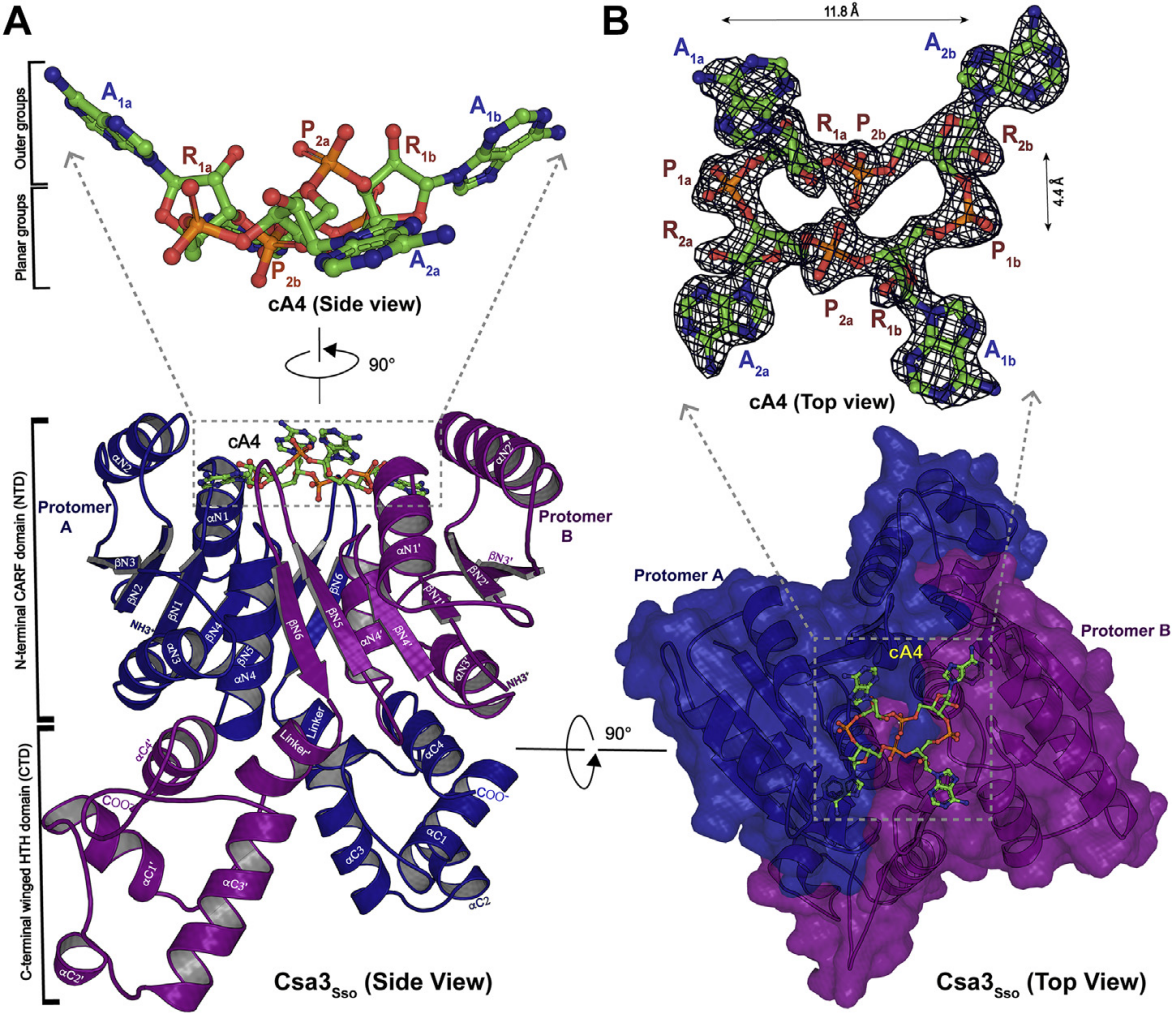
**X-ray crystal structure of Csa3**_**Sso**_**•cA4 complex at 2.05 Å.***A*, a side view of the X-ray crystal structure of Csa3_Sso_ dimer complexed with cA4 (ball-and-stick model) using its N-terminal CARF domain. *Blue* and *purple cartoons* represent two Csa3_Sso_ protomers in the dimer. The Csa3_Sso_ secondary structure elements are labeled with “type” (α or β), followed by “domain” (N or C terminal), and “number” (1–6 for β strands and 1–4 for α-helices). Protomer B elements are labeled with an apostrophe symbol (‘) to differentiate them from protomer A elements. αC3 is connected to αC4 by a flexible linker shown as a *dashed blue line*. The zoomed-in inset on the *top* shows an expanded side view of cA4 (ball-and-stick model), highlighting the “planar” and “outward facing” (outer) groups in cA4. *B*, a top view of the Csa3_Sso_•cA4 structure showing Csa3_Sso_ in the surface representation (*blue* and *purple*). To obtain this view of the Csa3_Sso_•cA4 model, the structure illustrated in A was rotated 90° in the direction indicated by the *black arrow*. The zoomed-in inset on the *top* shows an expanded top view of cA4 (ball-and-stick model), with a composite electron density map (2F_o_-F_c_, contoured at 2.0 σ) of cA4 in the refined Csa3_Sso_•cA4 structure. In both *A* and *B insets*, groups in cA4 are denoted as A, adenine; R, ribose; P, phosphoryl. To simplify illustration of the bound cA4 in this text, we refer to its AMP moieties as AMP_1a_, AMP_1b_, AMP_2a_, and AMP_2b_; the corresponding adenine rings as A_1a,_ A_1b_, A_2a_, and A_2b_; its ribose rings as R_1a,_ R_1b_, R_2a_, and R_2b;_ and the four phosphoryl groups as P_1a_ (connecting R_1a_ and R_2a_), P_2a_ (connecting R_1b_ and R_2a_), P_1b_ (connecting R_1b_ and R_2b_), and P_2b_ (connecting R_2b_ and R_1a_). All the cA4 atoms had a clash score of <0.7 except for C4 and O4 atoms of R_2b_, which was allowed at 1.15 Å to fit cA4 molecule in the electron density.

To better understand the structural basis for Csa3_Sso_ ligand specificity, we determined a 2.05-Å X-ray crystal structure of His_6_-Csa3_Sso_ bound to cA4 (Table 1). The overall Csa3_Sso_•cA4 structure is very similar to that of the previously determined 1.8 Å apo Csa3_Sso_ structure (PDB ID 2WTE, RMSDs of 0.735 Å over all atoms, Fig. S2) (61). Like the apo Csa3_Sso_ structure, the Csa3_Sso_ dimer is domain swapped with respect to an N-terminal CARF and the C-terminal wHTH DNA-binding domains of the two protomers A and B (Fig. 2). The N-terminal CARF domain in each protomer is composed of six mixed β-strands flanked by four α-helices. The first five β-strands (βN1-βN5) run parallel, whereas the last one (βN6) runs antiparallel and connects the CARF domain to a C-terminal wHTH DNA-binding domain (residues 145–212) through a linker (residues 133–144). The linker is composed of two turns of α-helices followed by four residues in an elongated conformation. The C-terminal wHTH DNA-binding domain contains a right-handed three-helix bundle formed by three helices (αC1–αC3) where αC2 and αC3 comprise the wHTH motif with αC3 (residues 173–186) constituting the DNA recognition helix. The wHTH of Csa3_Sso_ belongs to the widespread MarR-like wHTH fold in which the DNA recognition helix is followed by two β-strands to make a “wing” that follows an α-helix. Accordingly, the HTH wing in the His_6_-Csa3_Sso_•cA4 structure is composed of the αC3–αC4 loop that is followed by αC4. Although residues 192 to 196 at the tip of this wing are disordered in protomer A, the electron density for all the protomer B wing residues was observed likely due to their stabilization by contacts with a symmetry-related subunit (Fig. 2*A*).

**Table 1:** Structural data collection and refinement statistics

Data	Csa3Sso–cA4 complex
Protein Data Bank ID	6WXQ
Data collection	
Space group	P2_1_2_1_2
Cell dimension	
A, b, c (Å)	74.79, 118.83, 64.05
α = β = γ (Deg)	90
Wavelength (Å)	0.92009
Resolution (Å)	35.67 (2.09–2.05)
R_merge_ (%)	9.2 (37.1)
I/I(σ)	19.6 (5.9)
Completeness (%)	99.9 (97.9)
Multiplicity	8.0 (7.4)
Total number of observed reflections	69,259 (3464)
Unique reflections	36,695 (1810)
CC1/2 (%)	99.8 (92.3)
Solvent content (%)	53.9
Refinement	
R_work_/R_free_ (%)	16.0/20.52
RMS deviation	
Bond length (Å)	0.008
Bond angle (Deg)	0.998
Number of atoms	3767
Protein	3371
Ligand	112
Water	284
Ramachandran plot	
Favorable (%)	99.03
Additionally allowed (%)	0.97
Outlier (%)	0

R_work_ = Σ ∥F_o_|−|F_c_∥/Σ |F_o_|, calculated with a working set of reflections. R_free_ is R_work_ calculated with only the test set with 10% of reflections. Data for the highest resolution shell are given in parentheses. The structure was determined using single crystals.

### The Csa3_Sso_•cA4 complex exists as a dimer *in vitro*

**Figure 3:**
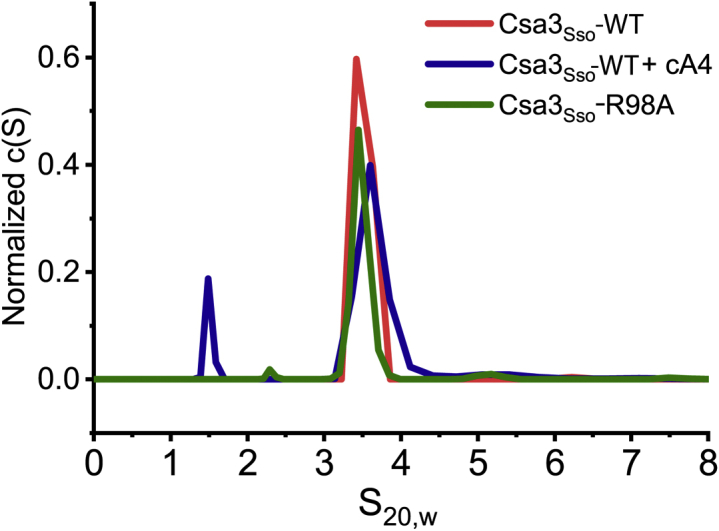
**cA4-bound Csa3**_**Sso**_**is a dimer in solution.** Sedimentation velocity analytical ultracentrifugation data showing *c(S)* distributions. *c(S)* values were derived from the fitting of the Lamm equation to the experimental data collected for wildtype Csa3_Sso_ (40 μM) in the absence (*red*) and presence of cA4 (50 μM) ligand (*blue*), as implemented in the program SEDFIT. The profile for the Csa3_Sso_-R98A mutant (64 μM) in the absence of cA4 ligand (*green*) is also shown. This analysis shows evidence of dimeric species in solution that persists upon the addition of cA4. The emergence of the 1.5S species in the presence of cA4 is interpreted to be mild protein degradation enduring during the time course of the experiment. Parameters derived from these analyses are presented in Table S1, and Lamm equation fits to the primary data are shown in Fig. S3.

Apo Csa3_Sso_ has previously been shown to exist in a dimeric form (61). However, crystal packing analysis of our Csa3_Sso_•cA4 crystal structure depicted dimer–dimer interactions that could underlie a higher-order oligomerization. More specifically, a symmetry mate obtained by operation X − 1/2, −Y + 1/2, −Z − 1 to the dimer in the asymmetric unit showed an increase in buried surface area of 2444.5 Å^2^ per dimer of Csa3_Sso_ using the PISA server (65, 66). Such a large crystallographic interface does not exist in the apo Csa3_Sso_ structure. To assess the possibility of cA4-induced Csa3_Sso_ oligomerization in solution, we performed size-exclusion chromatography. Consistent with prior reports, our Csa3_Sso_ preparations eluted as dimers in gel filtration chromatography (∼60 kDa *versus* globular standards, Fig. 1*C*). We further analyzed the oligomeric properties of Csa3_Sso_ in the presence and absence of cA4 using sedimentation velocity analytical ultracentrifugation (SV-AUC) (Figs. 3 and S3 and Table S1). In SV-AUC, His_6_-Csa3_Sso_ (96 μM) appeared as a single peak at 3.5 S_20,w_ with estimated mass (M_f_) of 52 kDa, consistent with the theoretically calculated S and mass values of 3.8 S and 52.3 kDa from the apo Csa3 structure (PDB 2WTE) (Figs. 3 and S3 and Table S1) (61). The addition of excess amounts of cA4 (Figs. 3 and S3 and Table S1) yielded a very similar dimer profile (3.6 S_20,w_ and 61.2 kDa), consistent with the calculated S and mass values from our Csa3_Sso_•cA4 structure with cA4 bound (3.86 S_20,w_ and 57.5 kDa). Overall, these data evidence monodisperse dimers of Csa3_Sso_ in solution that persist in the presence and absence of cA4. Consistent with in-solution dimers observed for Csa3_Sso_, the asymmetric unit for the Csa3_Sso_•cA4 complex structure consisted of a dimer of Csa3_Sso_ bound to one molecule of cA4 (Fig. 2*B*).

### Conformation adopted by cA_4_ in the Csa3_Sso_•cA4 structure

**Figure 4:**
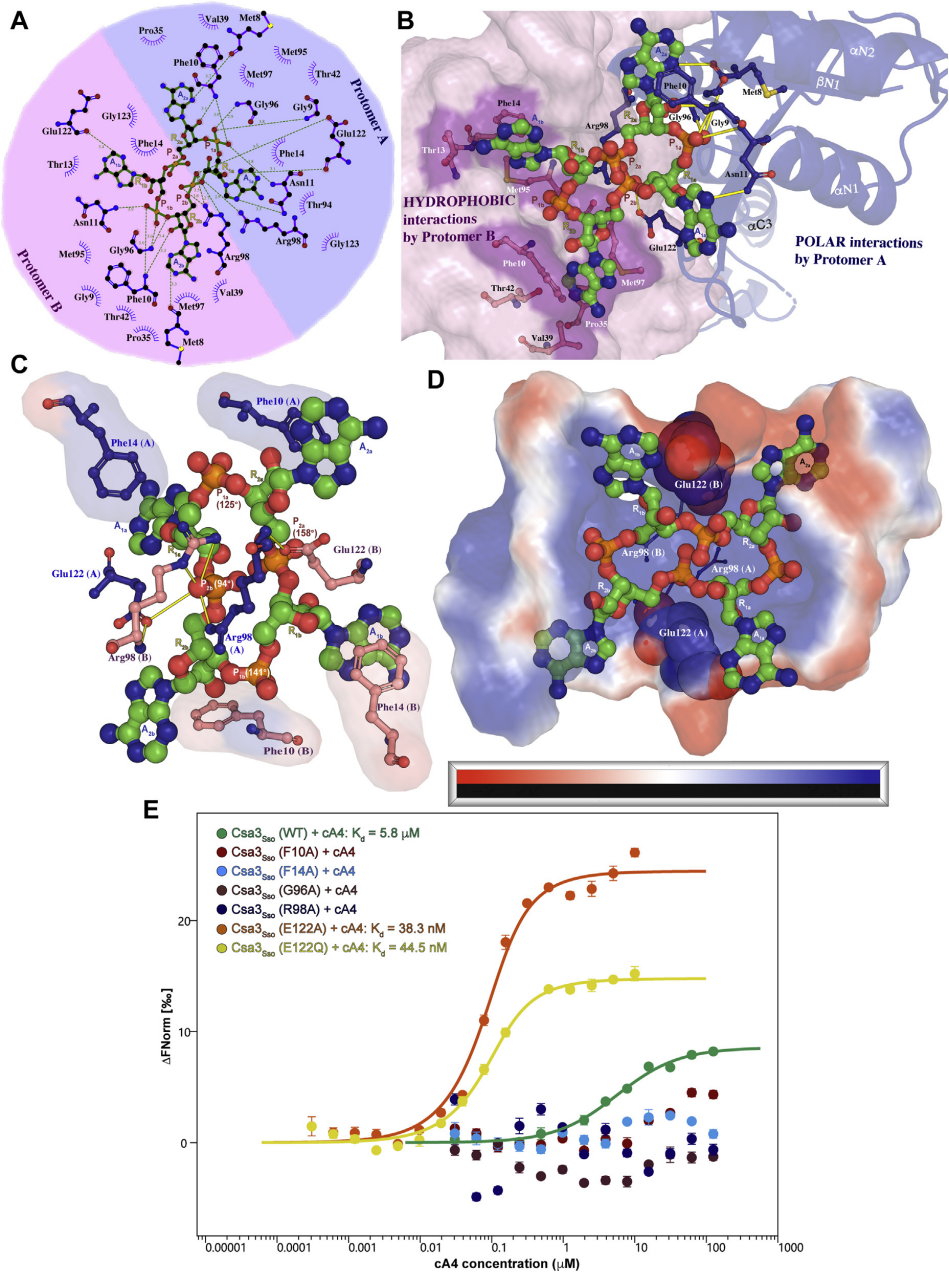
**Interactions of cA4 with Csa3**_**Sso**_**.***A* and *B*, symmetric interactions of the Csa3_Sso_ protomers A and B with the two halves of cA4. *A*, a LigPlot^+^ illustration of Csa3_Sso_ interactions with cA4 showing 2-fold symmetry in the binding pocket. The cA4 and interacting Csa3_Sso_ residues are shown in a ball-and-stick representation with *green*, *purple*, and *blue sticks* representing cA4, promoter A, and promoter B, respectively. To depict the symmetric orientation of cA4 and its interactions with Csa3_Sso_ residues with respect to CARF dimerization interface, the corresponding Csa3_Sso_ protomer regions are shown by *blue* (protomer A) and *purple* (protomer B) backgrounds. *B*, a top view of the Csa3_Sso_•cA4 complex structure showing interactions of cA4 with CARF domains from the two Csa3_Sso_ protomers. Csa3_Sso_ protomers A and B are depicted in *blue surface* and *purple cartoon* representations, respectively. For simplicity of illustration, only the hydrophobic interactions from protomer B (*purple residues* in ball-and-stick representation) and only the polar interactions from protomer A (*blue residues* in ball-and-stick representation) are shown. The hydrogen bonds are depicted as *solid yellow* (*B*) and *dashed green* (*A*) lines with interatomic distances labeled above the *green lines*. In both *A* and *B*, groups in cA4 are denoted as A, adenine; R, ribose; P, phosphoryl. *C*, a bottom-up view of the polar interactions of Csa3_Sso_-Arg98 and -E122 residues with P_2b_ phosphoryl oxygens in cA4. *D*, surface electrostatic potential distribution of the cA4-binding pocket showing electrostatic repulsion of the negatively charged central phosphoryl in cA4 by negative charge from Csa3_Sso_-E122 side chain. Calculations of surface electrostatic potential distribution were performed with APBS electrostatics plugin in Pymol program using default parameters. Electrostatic potential values are shown in a scale from *red* to *blue*, corresponding to −5.0 and +5.0 kcal/(mol), respectively, at 310 K. *E*, binding affinities of Csa3_Sso_ mutants targeting the Csa3_Sso_ structural interface with cA4 are measured using microscale thermophoresis. The graph displays data from three independent measurements. Error bars represent standard deviation.

Comparison of all the cA4-bound structures of CARF domain proteins revealed that cA4 in the Csa3_Sso_–cA_4_ complex structure exists in a unique elongated conformation. In this conformation, P_1a_ and P_1b_ (called *distal* phosphoryl groups) in the cA4 ring are stretched outward (with a P-to-P distance of 11.8 Å), which brings P_2a_ and P_2b_ (called *proximal* phosphoryl groups) to a shorter P-to-P distance of 4.4 Å (Fig. 2*B* inset). The difference of 7.4 Å in these P-to-P distances exemplifies the most extended conformation of cA4 observed among known cA4 receptors (Fig. S4). Furthermore, A_2a_ and A_2b_ adenines adopt a conformation parallel to the plane of cA4 backbone (hereafter called *planar adenines*), whereas A_1a_ and A_1b_ adenines face outside of this plane pointing away from the binding pocket (hereafter called *nonplanar adenines* [Fig. 2*A* inset]). Similarly, 2′ hydroxyls in R_1a_ and R_1b_ and both unbonded oxygens in P_2a_ face away from the binding pocket (Fig. 2*A* inset). Three of the phosphoryl groups in cA4 (labeled P_1a_, P_1b_, and P_2b_) are *planar*, whereas P_2a_ faces away from the binding pocket (hereafter called *nonplanar* phosphoryl). Furthermore, the 2′ hydroxyl oxygen (O’), phosphorous (P), and 5′- ribosyl oxygen (O’’) of R_1a_, R_1b_, R_2a_, and R_2b_ exhibit O’-P-O” angles of 125°, 141°, 158°, and 94°, respectively (Fig. 4*C*).

### cA_4_ recognition by Csa3_Sso_

**Figure 5:**
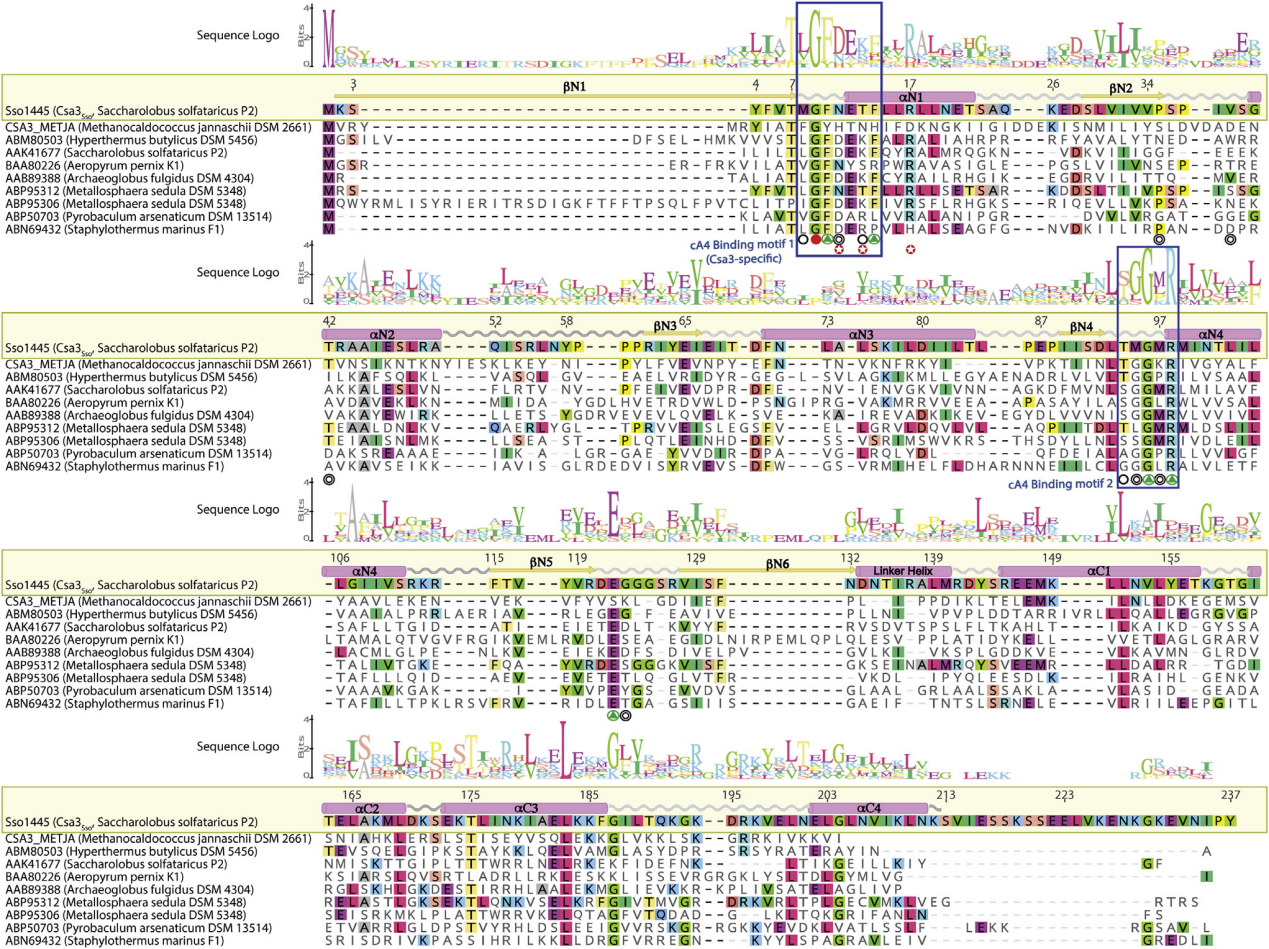
**Structure-guided sequence alignment of Csa3 homologs.** The Csa3_Sso_ residues at the Csa3_Sso_•cA4 structural interface are identified by *circles* below the alignment. The Csa3_Sso_ residues marked with *stars* at the *bottom* of alignment identify cA4 binding motif identified in the nuclease receptors of cOAs containing the CARF domain. *Blue rectangles* depict cA4 binding motifs 1 and 2 as determined from the Csa3_Sso_•cA4 structure. *Circles* filled with *green triangles* identify conserved (>65% similarity among ten Csa3 homologs) residues at the cA4 interfaces in both the Csa3_Sso_ protomers that showed significant loss or gain of function in the cA4 binding assay (Fig. 4*E*). *Empty circles* and those filled with *smaller circles* represent the interfacial residues present in one or both protomer(s) in the Csa3_Sso_ dimer, respectively, and were not subjected to mutagenesis. *Circles* filled with *red color* identify interfacial residues that are conserved but were not subjected to mutagenesis in this study. The numbering is based on the residue positions of the Csa3_Sso_ (gene accession number: Sso1445). The conservation of residues at each position is depicted by the size of the letters in the sequence logo on *top* of alignment, where the most conserved residues are highlighted by a larger-sized letter and a by a *black background*. Logo letters colored *blue*, *green*, *red*, and *black* indicate basic, polar, acidic, and hydrophobic residues, respectively. The secondary structure elements are derived from the Csa3_Sso_•cA4 structure (in which α-helices are shown as *magenta cylinders* and β-sheets are shown as *yellow arrows*). The sequence alignments of the Csa3 homologs were performed using the T-Coffee method (107) and were edited using Geneious Prime software (https://www.geneious.com) and Adobe Illustrator (version 25.3). Each homolog is identified by its accession number and bacterial source.

Superimposition of our cA4-bound structure with the apo-Csa3_Sso_ structure (PDB 2WTE) revealed that the cA4-binding pocket is largely preformed upon dimerization of the CARF domain (residues 1–132) (Fig. 2). Owing to symmetry in this pocket, overlapping sets of CARF residues from both the Csa3_Sso_ protomers make equivalent interactions with the two halves of cA4. More specifically, CARF residues from protomers A and B of the Csa3_Sso_ dimer (labeled with subscripts A and B in this text) interact almost exclusively with adenine-, ribose-, and terminal phosphoryl groups labeled “*a”* (for A_1a_, A_2a_, R_1a_, R_2a_, and P_1a_) and “*b”* (A_1b_, A_2b_, R_1b_, R_2b_, and P_1b_), respectively (Fig. 4, *A*–*D*). Our structural analysis of the Csa3_Sso_–cA4 interface as well as sequence alignment of ten archaeal Csa3_Sso_ homologs refined the two previously predicted ligand-binding motifs within the Csa3_Sso_ CARF domain (Figs. 4 and 5, also see Discussion) (61). The first motif (named *cA4 binding motif 1*) comprises residues 8 to 14 (from the β1–α1 loop and the α1 helix), and the second motif (named *cA4 binding motif 2*) comprises residues 94 to 99 (located in the β4–α4 loop and α4 helix). Residues from these motifs as well as other regions in Csa3_Sso_ make an extensive network of hydrogen bonding and hydrophobic interactions with different cA4 groups (Fig. 4, *A* and *B*) as described below.

#### Csa3_Sso_ interactions with cA4 adenine rings

Extensive hydrophobic interactions from residues in *cA4 binding motifs 1* and *2* stabilize all four cA4 adenine rings. The nonplanar A_1_ adenines (A_1a_ and A_1b_) dock into shallow hydrophobic pockets formed by Thr13_A&B_ and Phe14_A&B_ (from *cA4 binding motif 1*), whereas the planar A_2_ adenines (A_2a_ and A_2b_) occupy much elaborate and deep hydrophobic pockets. In the planar adenine pocket, one face of the adenine is stabilized by a fully conserved Phe10_A&B_ (*cA4 binding motif 1*) and the other face is docked onto lesser conserved Met97_A&B_ (*cA4 binding motif 2*), Pro35_A&B_, Val39_A&B_, Thr42_A&B_, and T13_B_ (Fig. 4, *A* and *B*). The hallmark of all the adenine-binding pockets in Csa3_Sso_ are π-stacking interactions of Phe14_A&B_ and Phe10_A&B_ with the nonplanar (A_1a_ and A_1b_) and planar (A_2a_ and A_2b_) adenine rings, respectively. More specifically, Phe14_A_ and Phe10_B_ stabilize adenines A_1a_ and A_2b_ by T-shaped π-stacking interactions, and sandwich-type π-stacking interactions from Phe14_B_ and Phe10_A_ engage A_1b_ and A_2a_, respectively (Fig. 4*C*). Both Phe14 and Phe10 are significantly conserved in Csa3_Sso_ homologs; Phe14 is conserved in six of the ten Csa3_Sso_ homologs, and Phe10 is conserved as a Phe or Tyr in all ten homologs (Fig. 5). Furthermore, structural superimposition of cA4-bound Csa3_Sso_ with apo Csa3_Sso_ shows linear movement of Phe14_A_ or 90° rotation of Phe14_B_ likely facilitating interactions with cA4 (data not shown). To analyze the contribution of Phe14 and Phe10 to cA4 binding, we mutagenized these residues to alanines and investigated their ability to bind cA4 by MST. The alanine mutants of both Phe14 and Phe10 showed a complete loss of cA4 binding *in vitro* highlighting their important role in cA4 stacking (Fig. 4*E*).

Among the polar interactions of Csa3_Sso_ with the cA4 adenines, side chains of Asn11_A&B_ hydrogen bond to N7 in the nonplanar adenines (A_1a_ and A_1b_). Since Asn11 is conserved as an Asn, Asp, or His in most of the Csa3 homologs (Fig. 5), it is conceivable that the side-chain carboxylic oxygen or ring nitrogen atoms in these homologs could instead hydrogen bond to the nonplanar adenines (Fig. 4, *A* and *B*). Furthermore, the main-chain oxygen atoms of Met8_A&B_ (*cA4 binding motif 1*) and Glu122_A&B_ (β5–β6 loop) also hydrogen bond N3 in the planar (A_2a_ and A_2b_) and nonplanar (A_1a_ and A_1b_) adenines, respectively (Fig. 4, *A* and *B*).

#### Csa3_Sso_ interactions with phosphate and ribose groups at distal ends of cA4

The P_1_ phosphoryls (P_1a_ and P_1b_) and R2 ribosyls (R_2a_ and R_2b_) at the distal ends of the elongated cA_4_ are hydrogen bonded by main-chain atoms of many residues in the *cA4 binding motifs 1* and *2* (Fig. 4, *A* and *B*). More specifically, the main-chain nitrogen atoms of Phe10_A&B_ hydrogen bond with 2′ ribosyl oxygens of R_2_ groups (R_2a_ and R_2b_) as well as with P_1_ phosphoryl oxygens (P_1a_ and P_1b_). The main-chain nitrogen atoms of Gly9_A_, Asn11_A&B_, and Gly96_A&B_ hydrogen bond with the P_1_ phosphoryl oxygens (P_1a_ and P_1b_). Among hydrophobic interactions, main-chain atoms of Gly9_B_, Gly96_A&B_, and Met95_A&B_ further stabilize the P_1_ and R_2_ groups. Of interest, Gly9 and Gly96 are parts of β1–α1 and β4–α4 loops in motifs 1 and 2, respectively, and are completely conserved among Csa3 homologs (Fig. 5). It is therefore possible that these glycines add flexibility to these loops to facilitate binding of the P_1_ phosphoryl groups in an extended cA4 conformation (Fig. S4). Furthermore, Csa3_Sso_-Met95 is conserved as a glycine in most of the Csa3 homologs, which may further be adding to the flexibility of the β4–α4 loop to accommodate this cA4 conformation (Fig. 5). To test whether small glycine residues play a role in binding, we mutagenized Gly96 to a slightly bulkier residue alanine. Indeed, the G96A mutant completely lost the ability to bind cA4 in our MST assay, confirming the requirement of a small residue at this position (Fig. 4*E*).

#### Csa3_Sso_ interactions with proximal cA4 phosphoryls

The inward-facing phosphoryl group in the middle of the elongated cA4 (P_2b_) was found to hydrogen bond with highly conserved Arg98_A&B_ (*cA4 binding motif 2*) and Glu122_A_ (β5–α5 loop) residues in Csa3_Sso_. The interactions with Arg98 involve hydrogen bonds to P_2b_ oxygens using either two (for Arg98_B_) or one (for Arg98_A_) of its side-chain nitrogen atoms (Fig. 4, *A* and *B*). Arg98 is completely conserved in all the Csa3_Sso_ homologs (Fig. 5). Accordingly, our alanine mutant of Arg98 completely lost cA4 binding *in vitro* (Fig. 4*E*). Since Arg98 also contributes significantly to dimerization interface of Csa3_Sso_ (buried surface area per Arg = 256.14 Å^2^), we confirmed that this mutant is still dimeric in solution using SV-AUC (Figs. 3 and S3 and Table S1).

Of interest, the cA4 phosphoryl interacting surface of the Csa3_Sso_ pocket is largely positively charged except for the conserved Glu122 residue that hydrogen bonds with cA4 central phosphoryl (P_2b_) oxygen *via* its side-chain carboxyl in the protomer A (Fig. 4, *A* and *B*). Such interactions of Glu122 side chains could create an electrostatic repulsion with central phosphoryls (P_2a_ and P_2b_) likely constraining them close to each other in the elongated cA4 conformation (Fig. 4*D*). The Glu122_A&B_ side-chain carboxyl also hydrogen bonds with the main-chain nitrogen of Arg98_B&A_ in the alternate protomer across the dimerization interface (Fig. 4*C*). Given the role of Arg98- and Gly96-containing motif 2 in cA4 binding discussed above, we wondered if Glu122 interactions with Arg98 main chain help position these motif 2 residues to affect cA4 binding. We therefore hypothesized that substitution of the Glu122 side chain to an Ala should remove electrostatic Glu122 repulsion to the central phosphoryls and disrupt polar interactions with motif 2, which should facilitate Csa3_Sso_ binding to cA4. Indeed, a mutation of Glu122 to alanine (E122A) dramatically improved the binding affinity of Csa3_Sso_ for cA4 (K_D_ of 38.3 nM, ∼145-fold higher affinity over wildtype) (Fig. 4*E*). To further dissect whether this cA4 binding gain is due to removal of side chain charge, or to the disruption of interactions with Arg98 main chain, we mutagenized Glu122 to a neutral Gln residue, which should still be able to make polar interactions with Arg98 (and P_2b_). An E122Q mutation was also found to drastically increase the cA4 binding affinity *in vitro* (K_D_ of 44.5 nM, ∼125-fold over wildtype) showing a more significant role of electrostatic repulsion by Glu122 as compared with its coordination of Arg98 (Fig. 4*E*). In summary, these results reveal the mechanistic basis underlying the inability of wildtype Csa3_Sso_ to strongly bind cA4 and further confirm that we have identified a biologically important Csa3 protein surface.

### Csa3_Sso_ lacks ring nuclease activity

**Figure 6:**
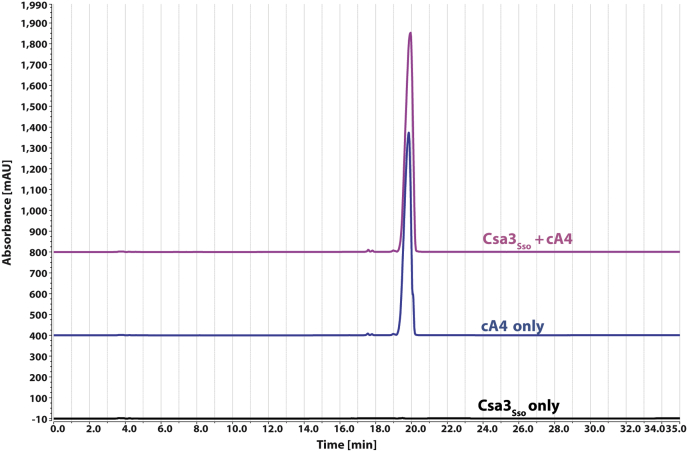
**Csa3**_**Sso**_**lacks ring nuclease activity.** No changes in the time-dependent stability of cA4 were observed in the reaction mixture containing 5 μM Csa3_Sso_ and 500 μM cA4 in buffer G (see Experimental procedures) at 60 °C for 3 h. Buffer G has previously been used for detecting the ring nuclease activity in Csm6 (84). The reactions were analyzed by C18 HPLC analysis after quenching and deproteination by phenol–chloroform extractions.

In the CARF-containing ring nucleases, the motif I is primarily implicated in the catalytic activity (67). Nevertheless, a conserved cOA-binding lysine in motif II of cA4 ring nucleases (Lys168 in Sso1393 and Lys106 in Sso2081) (67) was predicted to participate in cOA catalysis by stabilizing the transition state (68). Given Arg98_A&B_ coordination with P_2b_ at the end of the β4–α4 loop in Csa3_Sso_•cA4 structure, we tested cA4 ring nuclease activity of Csa3_Sso_ in two different conditions (Figs. 6 and S5, *A* and *B*). A C18 HPLC analysis of the reaction components after removal of Csa3_Sso_ showed no sign of cA4 hydrolysis products (Fig. 6). Consistent with this, P_2b_ exhibits an O’-P-O” angle of 94° in the Csa3_Sso_•cA4 structure, which is inconducive for an inline nucleophilic attack by the 2′ ribosyl hydroxyl.

In a few CARF-containing ring nucleases, polar interactions of a conserved Glu or Asp residue have been proposed to position their catalytic loop for activity (67). To further determine whether Glu122_A_ side chain coordination of Arg98 main chain and/or negative charge of Glu122_A_ side chain around central phosphoryl (P_2b_) prevents ring nuclease activity in Csa3_Sso_, we tested cA4 hydrolysis activity of the E122A and E122Q mutants, respectively. However, no ring nuclease activity was observed for these mutants (Fig. S5*C*). Overall, our Csa3_Sso_ data are consistent with the proposal that CARF motif 2 is involved in cA4 binding and not catalysis (67).

### Solution structure reveals cA4-induced wHTH domain rearrangements in Csa3_Sso_

**Figure 7:**
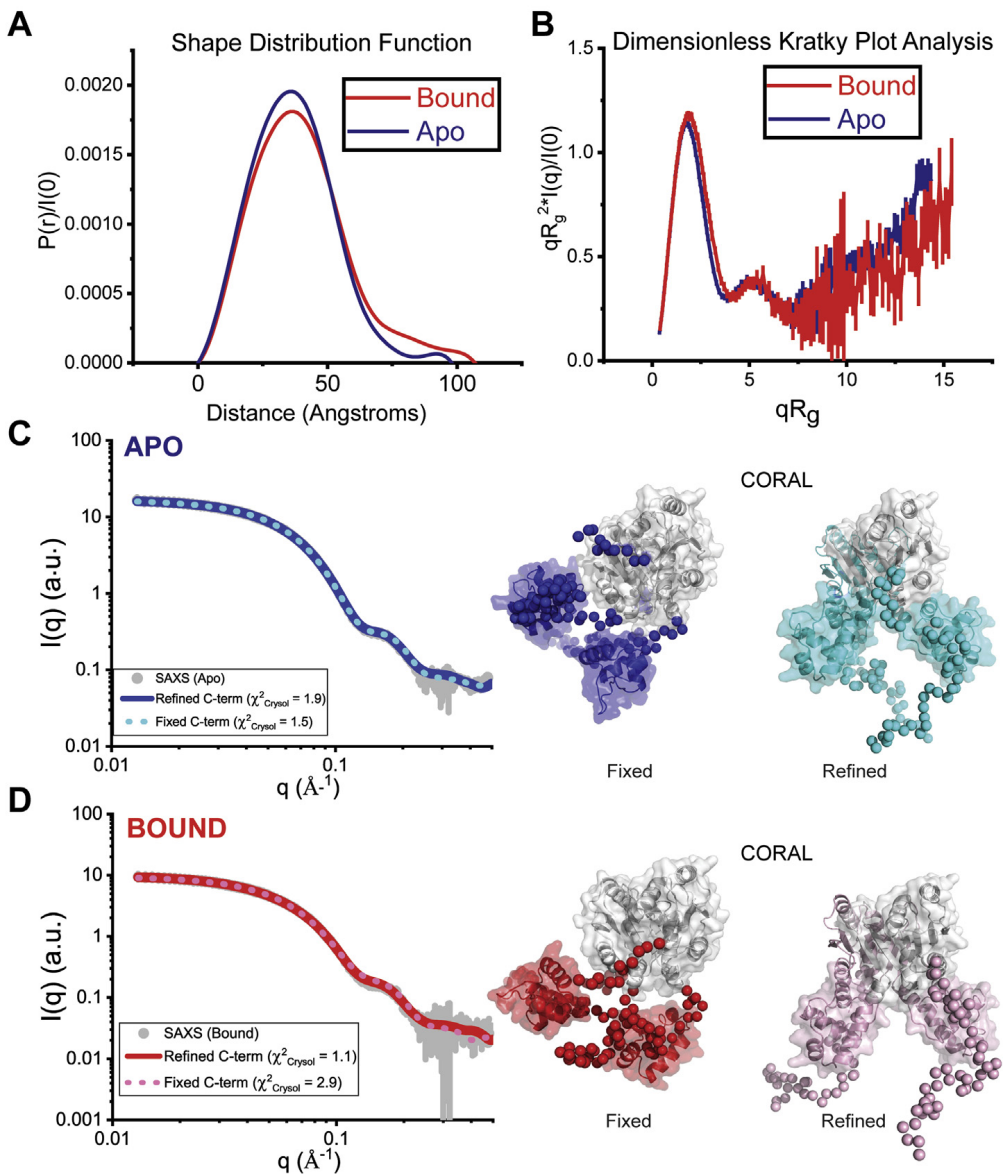
**SAXS analysis of cA4-induced conformational changes in Csa3**_**Sso**_**dimers.***A*, shape distribution function analysis for Apo (*blue line*) and cA4-bound (*red line*) Csa3_Sso_ dimers, performed using the program GNOM (101). Parameters derived from this analysis are provided in Table 2. In the bound form, a redistribution of interatomic vectors from ∼38 to ∼80 Å is observed, consistent with rearrangement of globular domains in response to a ligand. *B*, dimensionless Kratky Plot analysis (105), where the intensity of scattering is plotted as qR_g_^2^∗I(q)/I(0) *versus* qR_g_^2^. R_g_ is the radius of gyration in Å, I is the scattering intensity in arbitrary units, and q is the scattering angle (q = 4π sin(θ)/λ, where λ is the X-ray wavelength and 2θ is the scattering angle). Both Apo (*blue line*) and cA4-bound (*red line*) Csa3_Sso_ dimers show a characteristic bell-shaped peak at low-q that returns to near baseline at wider scattering angles, indicative of a more compact, globular macromolecule. *C* and *D*, CORAL analysis (70) of Csa3_Sso_ dimers, which employs a rigid body approach to optimize crystallographic models against experimental SAXS data. In this approach, missing and flexible atomic inventory are represented as beads in coarse grain fashion and flexibly fit. Shown on the *left* are the experimental SAXS data for both Apo (*C*, *blue*) and cA4-bound (*D*, *red*) as *gray circles* in a log–log plot, where intensity I is plotted as a function of q. In this analysis, two modeling approaches were considered in each state: a “fixed” approach, where the C-terminal wHTH domains were fixed in their crystallographic configuration, and a “refined” calculation, where the C-terminal domains were additionally refined in atomic position. In the Apo state (*C*), the fixed configuration (*cyan dotted line*) showed a slightly better agreement (χ^2^ = 1.5) than the calculations where wHTH positions were refined (*blue solid line*, χ^2^ = 1.9). Conversely, in the cA4-bound state (*D*), the fixed configuration (*pink dotted line*) showed worst agreement with the solution data (χ^2^ = 2.9) than the calculations where the wHTH positions were refined (*red solid line*, χ^2^ = 1.1). The corresponding structural models derived are shown to the *right* for both the apo and cA4-bound states. A gallery of representative calculations (n = 10) in each state is provided in Fig. S7.

The high overall similarity of the Csa3_Sso_•cA4 complex structure with the apo Csa3_Sso_ structure (61) suggested no significant Csa3_Sso_ conformational changes upon cA4 binding (Fig. S2). This was perplexing to us specially since conformational differences in Csa3_Sso_ could be the only other way to understand functional relevance of the cA4 binding given cA4 did not change the oligomerization state of Csa3_Sso_ in our SV-AUC experiments. Also, cA4 binding has been previously shown to induce conformational changes in Can1 nuclease receptor of cA4 (69). To determine if the Csa3_Sso_•cA4 crystal structure accurately represents the solution state of this complex, we conducted small-angle X-ray scattering (SAXS) analysis of Csa3_Sso_ in the presence and absence of cA4. In the presence of excess cA4 (sufficient to saturate all cA4-binding sites), the shape of dimeric Csa3_Sso_ changed significantly. The data for Csa3_Sso_ both in the presence and absence of cA4 displayed linearity in the classical Guinier analysis (Fig. S6), with an observed increase in the radius of gyration (R_g_) in both the Guinier and inverse Fourier transform (GNOM) analyses (Fig. 7 and Table 2). Mass calculations from this data are consistent with in-solution dimers from both the states (Table 2). By Pr analysis, these differences coincide with increases in R_g_ and D_max_ and a redistribution of interatomic vectors to greater values (Fig. 7*A*). The numerical values derived from these analyses correlate very well with SAXS measurements made previously for apo Csa3_Sso_ (61). This suggested that there is a significant conformational difference between the bound and apo states of Csa3_Sso_ in solution.

**Table 2:** Parameters derived from SAXS analysis of Csa3_Sso_

Sample	Concentration (mg/ml)	Guinier		GNOM		P_x_^a^	MM (kDa)^b^
		qRg	Rg (Å)^c^	Rg (Å)	Dmax (Å)		
Csa3_Sso_	8.3	0.38–1.23	28.7 ± 0.1	28.2 ± 0.1	100	3.9	58.9 (56)
	6.7	0.23–1.28	28.4 ± 0.1	28.1 ± 0.2	101	3.9	58.9 (56)
Csa3_Sso_ + 180 μM cA4	5.5	0.40–1.26	30.8 ± 0.2	30.9 ± 0.2	110	4.0	66.1 (56)
	4.4	0.44–1.34	31.3 ± 0.2	31.2 ± 0.3	110	4.0	66.4 (56)

a Porod exponent (P_x_). Values near ∼4 indicate compactness, whereas lower values between 2 and 3 indicate significant lack of compactness and increased volumes (105). These values were determined using the program ScÅtter (https://bl1231.als.lbl.gov/scatter/).

b Mass determinations using the Q_r_ invariant (106) were determined using the program RAW. Expected dimeric mass is shown in parentheses.

c Errors reported reflect the uncertainty in the value for R_g_ determined using classical Guinier fitting.

Model-independent analyses including Guinier, Kratky, Porod-Debye, and mass calculations indicate that the cA4-induced conformation of the dimer is not due to changes in flexibility and disorder, or mass, but rather discrete differences in the configurations of the structural domains (Figs. 7*B* and S6 and Table 2). Although low in resolution, SAXS analysis allows for the rigorous testing of atomic models against their solution properties. These model-independent analyses would indicate that single atomistic models (*ab initio* or atomistic) could be reliably tested against the solution data to discern the nature of these conformational changes.

**Figure 8:**
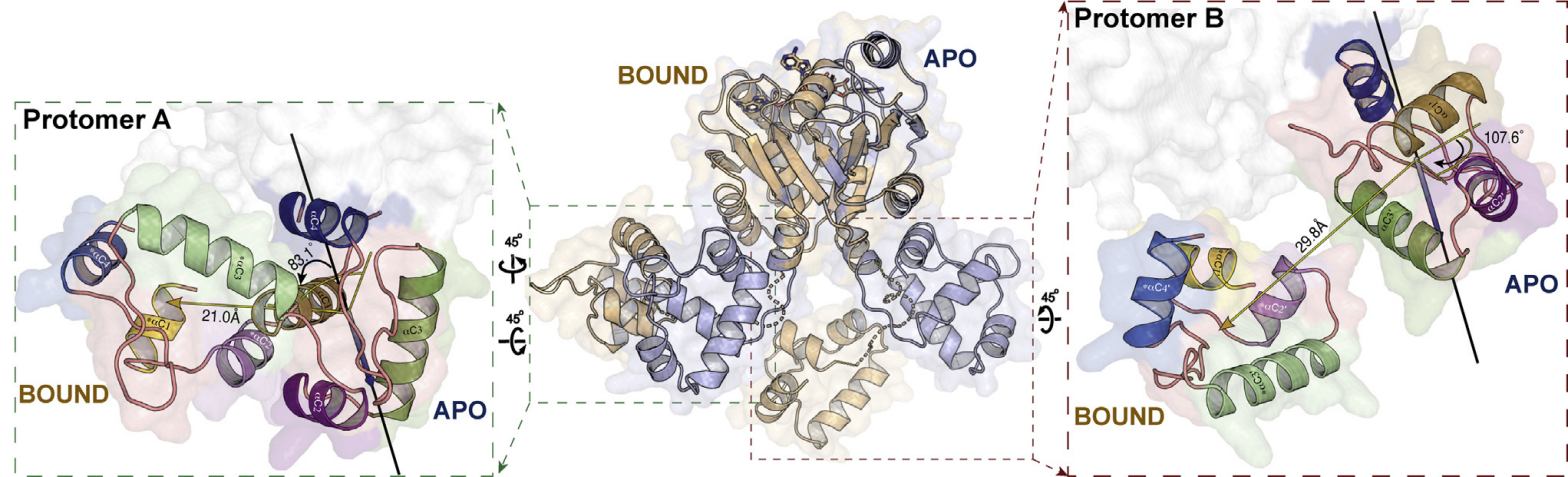
**Comparison of apo and cA4-bound structures of Csa3**_**Sso**_**dimers in solution.** The central panel depicts superimposition of the apo (*blue cartoon*) and cA4-bound (*brown cartoon*) conformations of the Csa3_Sso_ dimer, and shows overall differences in the CARF-relative positions of the wHTH domains. The inset on the left (outlined with *green dotted lines*) illustrates conformational change in Protomer A wHTH that involves a rotation (shown as *yellow lines* along the *solid black-line* axis) and displacement (*yellow arrow*) of 83.1° and 21.0 Å, respectively. The inset on the *right* (outlined with *red dotted lines*) shows conformational change in Protomer B wHTH involving a rotation (shown as *yellow lines* along the *solid black-line* axis) and displacement (*yellow arrow*) of 107.6° and 29.8 Å, respectively. Views in the *left* and *right insets* were obtained by 45° rotations as indicated. Secondary structure elements in both insets are labeled following the scheme in Figure 2 with an inclusion of an *asterisk* (∗) prefix for the elements for cA4-bound state.

Crystallographic Csa3_Sso_ models only provide atomic inventory for 212 of the 248 a.a. in the His_6_-tagged Csa3_Sso_ construct used in this study, including the disordered C terminus (∼25 a.a.). To model this missing atomic inventory against its solution data, we employed the CORAL program, which uses coarse-grain beads for missing amino acids in a hybrid bead-atomistic modeling approach (70). When the method was applied, Csa3_Sso_ in its unbound form could be readily reconciled against the SAXS data (χ_Crysol_ = 1.5). No improvement was observed when allowing for the C-terminal wHTH domain positions to be refined *via* rigid-body docking, without symmetry constraints (Fig. 7*C*). In contrast, a significant preference was shown for a large asymmetric wHTH domain positioning over its symmetric crystallographic configuration in the cA4-bound state (χ_Crysol_ = 2.9 *versus* 1.1) (Fig. 7*D*). Therefore, the binding of cA4 to the Csa3_Sso_ dimer was found to induce large asymmetric conformational changes in the position of the two wHTH domains in solution (Fig. 8). These in-solution conformational changes involved asymmetric, but significant, rotations and displacements of the wHTH domains from chains A (wHTH_A_) and B (wHTH_B_) (Fig. 8). More specifically, the wHTH_A_ domain exhibited an 83.1° rotation and 20.9 Å displacement (left inset in Fig. 8), whereas the wHTH_B_ domain exhibited a 107.6° rotation and 29.8 Å displacement (right inset in Fig. 8). This results in what we refer to as a “closed” Csa3_Sso_ state. We envisage that Csa3_Sso_ samples between this closed and open (equivalent to apo conformation) states. Although it is possible that apo Csa3_Sso_ populates a complex population of open and closed states, the closed conformation is stabilized upon cA4 binding. Overall, our data demonstrate significant allosteric repositioning of wHTH_A_ and wHTH_B_ domains upon cA4 binding, which may underlie cA4 regulation of Csa3 signaling.

### cA4 does not affect specific DNA binding by Csa3_Sso_

**Figure 9:**
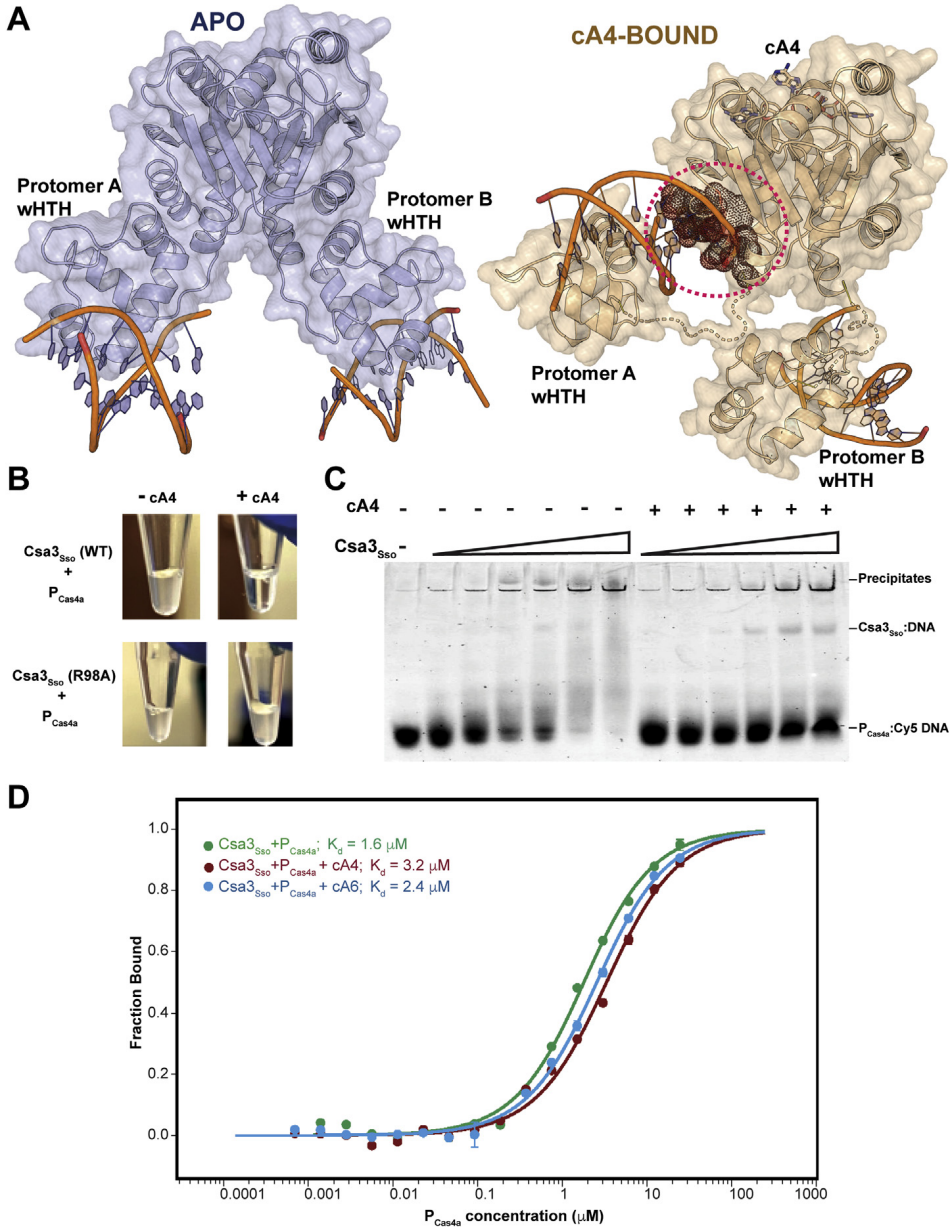
**cA4 does not significantly affect specific DNA binding by Csa3**_**Sso**_***in vitro*.***A*, predictive docking of DNA fragments (*orange backbone*) onto the wHTH domains of SAXS-derived *apo* (blue protein model on *left*) and cA4-bound (light brown protein model on *right*) Csa3_Sso_ dimers. The DNA fragment docked onto protomer A in the cA4-bound Csa3_Sso_ dimer sterically clashed with a region in the CARF domain from protomer B in this dimer (encircled by a *red dotted circle*). The DNA fragment docked onto protomer B in this cA4-bound dimer exhibited a ∼90° rotation instead. To obtain these models, wHTH domains of *B. subtilis* OhrR (PDB code: 1Z9C (71), RMSD: 1.7 Å) were individually superimposed onto wHTH domains of the Csa3_Sso_ dimer and OhrR was omitted from the view for clarity. A single and straight model of B-form DNA with two copies of the palindromes could not be modeled with the *apo* Csa3_Sso_ dimer owing to a slight misorientation (not shown) between Csa3_Sso_ wHTH domains (possibly requiring a bend in the DNA). *B*, precipitation of Csa3_Sso_ in the presence of P_Cas4a_ DNA and its solubilization by cA4 binding to the Csa3_Sso_ CARF domain. Before taking the photograph, 50 μM Csa3_Sso_ (WT or R98A mutant), 10 μM P_Cas4a_, and/or 500 μM cA4 were added in the binding buffer without Tween 20 (see Experimental procedures) and incubated for 30 min at 20 °C. *C* and *D*, cA4 does not significantly affect the binding of Csa3_Sso_ to the P_Cas4a_ promoter *in vitro*. EMSA shows no effect of cA4 on the specific binding of Csa3_Sso_ to P_Cas4a_ (*C*) that drives spacer acquisition in *S. solfataricus* P2. Cy5-labeled P_Cas4a_, 50 nM, was added to different Csa3_Sso_ concentrations ranging from 500 nM to 25 μM in the presence or absence of cA4 added in a 50:1 ratio of cA4:Csa3_Sso_. *D*, microscale thermophoresis binding affinity analysis also shows no significant effect of cA4 (as well as cA6) on the Csa3_Sso_ binding to the P_Cas4a_. For microscale thermophoresis, Csa3_Sso_ was held constant at 500 nM, and P_Cas4a_ concentration was varied from 763 nM to 25 μM in the absence or presence of 50-fold excess of cOAs (25 μM) to Csa3_Sso_.

Csa3a_Sis_ (a Csa3_Sso_ ortholog from *S. islandicus* REY15A) has been shown by Liu *et al.* to regulate transcription by binding to a pseudopalindromic consensus region upstream of CRISPR leader and spacer acquisition operon (*viz*., *cas1*, *cas2*, *cas4*, and *csa1*/*cas4a* genes) (59, 60). We therefore evaluated the effect of the cA4-induced repositioning of wHTHs observed in our cA4-bound solution structure on the DNA binding by Csa3_Sso_. Our superpositional docking of the DNA fragments from homologous OhrR–DNA complexes (PDB ID: 1Z9C) (71) onto these apo state structures showed no change in DNA conformations (Fig. 9*A* left panel) (61). However, the “closed-state” (cA4-bound) models exhibited drastically different docked DNA conformations for both wHTH_A_ and wHTH_B_. More specifically, the fragment docked onto the wHTH_A_ in the closed state sterically clashed with the CARF_B_, and the one docked onto wHTH_B_ showed an ∼90° rotation with respect to its position in the *apo* state model (Fig. 9*A* right panel). Since each docked DNA fragment here represents one of the two palindromes Csa3_Sso_ is expected to bind, we considered experimental evaluation of a possibility of DNA binding disruption by cA4 binding to Csa3_Sso_. For this, we performed initial electromobility shift assays (EMSAs) using the *cas4a* (accession ID: *sso1451*) promoter fragment (P_Cas4a_) originally predicted by Liu *et al.* (59) in *S. solfataricus* P2 genome to conserve the Csa3a-binding site (Fig. S8, *A* and *B*). Our negative controls lacking predicted Csa3-binding sites included a similarly long *leader A* as well as a DNA fragment unrelated to CRISPR systems (Fig. S8). Unexpectedly, Csa3_Sso_ interacted with all these fragments in a sequence-independent way generating protein–DNA precipitates that did not enter the gel (Fig. S8*C*). Furthermore, crude visualization of these Csa3_Sso_–P_Cas4a_ complex precipitates in microcentrifuge tubes showed that presence of excess of cA4 prevented their formation (Fig. 9*B*). To analyze if this cA4-mediated effect is due to a possible alteration in the physicochemical composition of the binding reaction by cA4, we used cA4 binding-deficient Csa3_Sso_-R98A mutant in this examination and found that cA4 could not rescue the Csa3_Sso_-R98A mutant from precipitating with the DNA (Fig. 9*B*). To analyze the effect of cA4 addition on the sequence-specific DNA binding by Csa3_Sso_, we chose P_Cas4a_ that additionally revealed a small proportion of the shifted probe representing soluble protein–DNA complexes (Fig. S8*C*). Surprisingly, however, we did not see a significant effect of cA4 on the levels of the minor soluble Csa3_Sso_–DNA complex population (faint shifted band), even using a more sensitive EMSA utilizing a Cy5-labeled P_Cas4a_ probe. However, the levels of the insoluble complex population were reduced in a cA4 concentration-dependent manner as indicated by an increase in the amount of the free probe in lanes with excess cA4 added (Fig. 9*C*).

To further analyze whether binding of cA4 affected specific DNA binding by Csa3_Sso_, we performed MST-based binding affinity analysis of Csa3_Sso_ using P_Cas4a_ as a ligand where we removed precipitated material before the fluorescence measurements. This analysis depicted a K_D_ of 1.62 ± 0.17 μM for binding of Csa3_Sso_ to P_Cas4a_, which is moderately better than that observed previously for Csa3b_Sis_ binding to an analogous *S. islandicus* P_Cas4a_ promoter (57). However, the addition of excess of cA4 (or cA6) did not significantly change the P_Cas4a_ binding affinity (K_D_s of 3.20 ± 0.36 and 2.4 ± 0.17 μM in the presence of cA4 or cA6, respectively) (Fig. 9*D*).

In conclusion, these analyses showed that binding of cA4 increases solubility of the Csa3_Sso_ in the presence of DNA. However, in our assays using the selected binding regions, the specific DNA binding affinity of Csa3_Sso_ was unaltered *in vitro* by adding cA4 alone. Although we might have missed the physiologically relevant DNA target of Csa3_Sso_, this also brings up possibilities of alternate models of transcriptional regulation discussed below.

## Discussion

The type I interference complexes are highly efficient in clearing infections associated with phage genomes containing an intact PAM sequence (72). The phages, on the other hand, have evolved escape strategies like the generation of escape mutations in the PAM sequences (1, 48, 73, 74) and production of anti-CRISPR (Acr) proteins (75, 76). During infection by a resilient phage, a coexistent type III interference (csm/cmr) complex works independent of the type I Acr- and PAM motif to facilitate phage clearance (Fig. 1) (25, 48, 49, 77, 78). However, excessive activity of the csm/cmr complexes and cOA receptors results in nonspecific genomic mutagenesis and RNA hydrolysis, respectively (54, 79). The type III interference therefore needs to prevent this aberrant fate during reinfection by the same virus. Consistent with this, the type III interference is bioinformatically predicted to trigger *de novo* spacer acquisition and crRNA production enabling the type I system for a future reinfection (Fig. 1, *A* and *B*) (4, 25). However, the regulatory mechanisms underlying such a reversal to type I interference are not clear.

A CARF domain is a variant of the Rossmann fold lacking the canonical G-X-G-X-(G/A) motif involved in binding NAD(P)H or FADH_2_ (80). Instead, CARF domains generally conserve a (D/N)-X-(S/T)-X_3_-(R/K) motif in their βN4–αN4 loop that is known to bind cOAs in many nucleic acid hydrolases (81). The βN4–αN4 loop is conserved in the Csa3 transcription factors (residues marked with white stars in red background in Fig. 5), which, along with the βN1–αN1 and βN5–βN6 loops, creates a potential nucleotide-binding pocket (61). However, biochemical and structural characterization of ligand binding specificity and regulation of Csa3 family transcriptional factors has been missing (4, 57, 61). Despite the low micromolar binding affinity we observed for cA4 binding to Csa3, we expect it to be physiologically relevant since high micromolar cA4 concentrations have been estimated to be attained in an infected *Sulfolobus* cell. More specifically, every phage transcript molecule detected by the type III interference complex produces an intracellular cA4 concentration of ∼6 μM. Thus, a concomitant synthesis of multiple phage RNA molecules could result in an intracellular cA4 concentration in the multiples of six to the number of RNA molecules detected (64). Therefore, a relevant biological scenario includes failure of type I CRISPR systems leading to the concomitant detection of several phage transcripts by the type III interference complexes, which then produce cA4 at a rate that significantly exceeds its hydrolysis by the ring nuclease effectors. As a low-affinity cA4 receptor, Csa3_Sso_ may therefore act only as a last resort to regulate spacer acquisition, crRNA synthesis, and DNA repair. Such a mechanism could help alleviate cellular toxicity known to increase upon extensive spacer acquisition (37). This is further supported by the fact that Csa3_Sso_ lacks any demonstratable ring nuclease activity *in vitro* (discussed below), which could reduce the effectiveness of such a system.

The previously predicted nucleotide-binding pocket in Csa3_Sso_ involved 12 CARF domain residues. Seven of these constitute the motif 1 (T_7_-Φ_8_-G_9_-F_10_-(D/N)_11_-E_12_-X4-R_17_), which, along with E_122_, forms two symmetry-related pocket walls, and the other four comprise motif 2 (L_93_-X2-G_96_-Φ_97_-R_98_), which serves as the floor of this pocket (Φ stands for a hydrophobic residue) (61). Of the 12 residues from these regions, eight were found at the Csa3_Sso_•cA4 structural interface, whereas four (namely, Thr7, Glu12, Arg17, and Leu93) were present outside of the interface (Figs. 4 and 5). Furthermore, we identified six additional Csa3_Sso_ residues at this interface (Thr13, Phe14, Pro35, Val39, Thr42, and Gly123). Of these, Phe14 is significantly conserved among Csa3 homologs (Fig. 5) and, along with Phe10 from motif 1, contributes substantially to hydrophobic stacking of cA4 adenines (Fig. 4*C*). Similar π–π stacking contributions of a Trp residue (Trp42) in a *Thermus thermophilus* Can1 receptor of cA4 have previously been proposed to stabilize cA4 in an asymmetrical conformation (69), which may also be true for the extended cA4 conformation bound to Csa3_Sso_. Furthermore, the essential interactions of the highly conserved Csa3_Sso_-Gly96 with the terminal cA4 phosphoryl moieties may additionally contribute to this stabilization (Fig. 4). Finally, our observed contribution of Csa3_Sso_-Arg98 interactions with the central phosphoryl moieties in cA4 is consistent with a previously reported loss of the binding of the cA4 analog to a multisite Csa3b_Sis_ mutant encompassing an Csa3_Sso_-Arg98-equivalent residue (57). These residues' functional relevance in Csa3a and Csa3b homologs for binding cA4 further confirms the validity of our observed Csa3_Sso_•cA4 structural interface to both these homologs. Therefore, based on our structure–function analysis, we propose following revised motifs facilitating cA4 binding to Csa3 proteins: Φ_8_-G_9_-F_10_-(N/D)_11_-X1-ζ_13_-F_14_ (cA4 binding motif 1) and π_94_-(π/Φ)_95_-G_96_-Φ_97_-R_98_) (cA4 binding motif 2), where subscripted numbers denote residue positions in Csa3_Sso_ and the symbols ζ, π, and X denote hydrophilic, small, and noninteracting amino acids, respectively (Fig. 5) (82). The cA4 binding motif 2 interacts primarily with the phosphoryl groups, whereas the cA4 binding motif 1 engages all the adenine rings in cA4 (Fig. 4, *A* and *B*). Of interest, the cA4 binding motif 2 is also conserved in other CARF proteins including Csx1, Csm6, and SiRe_0244 (a ring nuclease) (61, 68, 83, 84), whereas the cA4 binding motif 1 is unique to the Csa3 homologs (Fig. 5).

A conserved Glu122 from the βN5–βN6 loop (outside of the above two motifs) was found to significantly limit the cA4 binding affinity in Csa3_Sso_ *via* electrostatic repulsion to the central cA4 phosphoryls; substituting Glu122 with an Ala, or more conservatively with a Gln residue, drastically increased cA4 binding affinity of Csa3_Sso_ (∼145- or 125-fold, respectively) (Figs. 4 and 5). The conservation of Glu122 during evolution further indicates that it might be limiting Csa3 response only to high cellular cA4 concentrations during type III phage interference. This is also supported by the lack of a self-limiting ring nuclease activity in Csa3_Sso_. Nevertheless, this represents an interesting example of a rational surface design that increases ligand binding affinity and has potential applications in engineering an amplified Csa3 response to phage infections.

Owing to variation in the identity of the catalytic residues in the active sites of the known ring nucleases, the mechanistic details of ring nuclease activity are still emerging (67). Except for Crn3 that requires metal ions for ring nuclease activity, most of the cOA receptors employ a metal-independent nucleophilic substitution mechanism where a general base deprotonates a ribosyl 2′ hydroxyl (attacking group) for a nucleophilic attack on the scissile phosphorous atom and/or stabilizes a pentacovalent transition state by coordinating scissile phosphoryl oxygens (68, 85). Ultimately, these interactions position the 2′ hydroxyl oxygen (O’), phosphorous (P), and 5′- ribosyl oxygen (O’’) in-line (with an ideal O’-P-O” angle ∼180°) for the phosphodiester bond hydrolysis (86, 87). *Enterococcus italicus* cA6 ring nuclease (EiCsm6), however, is an exception where alternate residues in the protein sterically force cA6 O’-P-O” in a compatible in-line conformation (88). Although originally postulated to be involved in catalysis, the motif 2 Arg/Lys residues from CARF-containing ring nucleases have recently been found only to mediate cOA binding (67, 85). Instead, residues in another conserved motif GxS/T have been recently implicated in the catalysis, where Ser/Thr and a Trp residue conserved adjacent to this motif participate in the catalysis (68, 83, 84, 85, 88). Furthermore, a Glu or Asp residue conserved in a few CARF domain proteins coordinates the Gly to position the GxS/T motif adjacent to the scissile phosphoryl group, which is hypothesized to facilitate hydrolysis (67). Despite the essential interactions of the central cA4 phosphoryl (P_2b_) with the motif 2 residues in Csa3_Sso_ (Gly96 and Arg98), it lacks a demonstratable cA4 ring nuclease activity (Fig. 6). This could be due, at least in part, to the absence of the GxS/T motif in Csa3 proteins. Also, the active site interactions of Arg98 and Glu122 with the target cA4 phosphoryl (P_2b_) results in an O’-P’-O” angle of 94° presenting with a stereochemistry inconducive for nucleophilic attack by the 2′ ribosyl hydroxyl (Fig. 4*C*). By contrast, the nonplanar (P_2a_) and distal phosphates (P_1a_ and P_1b_) that have O’-P’-O” angles of 158°, 125°, and 141°, respectively, lack coordination by side chain of a basic residue to stabilize the transition state. The lack of ring nuclease activity in the Csa3_Sso_ proteins, unlike most self-limiting cOA ring nucleases, could allow for a long-term potentiation of the cA4 signal in an infected cell.

It is intriguing why X-ray crystallography data did not depict cA4-induced conformational changes revealed by our SAXS analysis. This could be explained, at least in part, by immobilization of the wHTH_B_ by its symmetry-related crystallographic interactions with CARF_A&B_ from the next Csa3_Sso_ dimer in our Csa3_Sso_•cA4 complex crystals. A significantly large interface constituted by these packing interactions could have driven selection of biologically infrequent Csa3_Sso_ conformer we observed in the crystal structure (Fig. S9).

Csa3b_Sis_ (a Csa3b homolog) regulation of type I interference (*cas*) genes and CRISPR spacer acquisition complex has been recently studied (57, 58). In the absence of an MGE, the subtype I-A interference (*cas*) genes are kept repressed by Csa3b_Sis_-mediated recruitment of the Cascade–crRNA complex at the P_Cas_ promoter. During MGE invasion, recognition of a protospacer sequence by the Cascade–crRNA complex facilitates its release from the P_Cas_ resulting in derepression of the *cas* gene expression (58). Furthermore, ∼10-fold increase in Csa3b_Sis_ binding affinity to the P_csa1_ promoter has been reported in the presence of the cA4 analog *in vitro*, which suggests a further repression of the adaptation gene expression upon phage transcript clearance by the type III interference complex (57). cA4-induced conformational changes we observed using SAXS bring DNA binding face of the wHTH_A_ (specifically helix αC3 that is expected to dock into the major groove of DNA) in proximity to the CARF domain (αN3 and βN2) from chain B (Fig. 8 left panel). Although this could potentially disrupt Csa3_Sso_ interactions with at least one copy of the two palindromes in its binding site (Fig. 9 right panel), our attempts to analyze the effects of cA4 on the binding of Csa3_Sso_ to P_Cas4a_ promoter showed no significant change in specific DNA binding by Csa3_Sso_. Although this unexpected observation does not fully align with previously published transcriptional regulation of the acquisition operon in *S. islandicus* by Csa3a_Sis_, it could simply be because the two Csa3a orthologs utilize alternate regulatory mechanisms. For example, cA4 binding could improve stability of DNA-bound Csa3_Sso_ or alter its DNA binding mode *in vivo*, which is supported by our increased Csa3_Sso_–DNA complex solubility *in vitro*. Alternatively, Csa3_Sso_ may need to interact with other similar ligand(s) or novel protein partner(s) for its transcriptional regulation. The latter mechanism, however, would be more intricate and distinct from a more common and straightforward regulatory model where ligand binding alone regulates DNA binding affinity of transcription factors to recruit RNA polymerase. Such an alternate mechanism could further harness different binding partners to differentially regulate a vast variety of CRISPR loci that *S. solfataricus* genome encodes in comparison with *S. islandicus*. Need for a Csa3_Sso_ partner is also indicated by *in vitro* instability of the Csa3_Sso_–DNA complexes we observed in the absence of cA4 *in vitro* (Figs. 9, *B* and *C* and S8*C*). Consistent with this, Csa3a proteins have been hypothesized to recruit transcription factor B to a noncanonical TATA box coexistent with the Csa3a promoters for spacer acquisition in *Sulfolobales* (59). Furthermore, such a cooperative interaction of Csa3_Sso_ with protein partners may also further improve Csa3_Sso_ binding affinities with cA4 and/or target DNA. Although we anticipate involvement of our observed cA4-induced Csa3_Sso_ conformations to underlie any sort of functional regulation, an additional possibility of DNA-dependent oligomerization cannot be ruled out with the existing data. Therefore, more work needs to be done to appreciate the binding of cA4 to Csa3_Sso_.

Finally, although possibilities always exist for other high-affinity ligands for Csa3 (that could also better regulate DNA binding by Csa3 *in vitro*), it is unlikely for a Csa3 CARF domain dimer with four binding pockets aptly engaged in recognizing a four-nucleotide ligand to accommodate a completely different ligand structure. In this context, it is of high interest to characterize Csa3a homologs from organisms that are reported to lack Cas10 and CRISPR-Cas loci to identify such possible alternate Csa3a ligands (67). Nonetheless, our data and coevolution of *csa3* genes with CRISPR loci in most prokaryotes support a cross talk between the type III and type I CRISPR systems in the form of cA4 binding to Csa3a homologs. This may underlie the regulation of spacer acquisition, crRNA gene expression, and (type I) Cascade -mediated clearance of re-infection by the same virus (Fig. 1*B*).

## Experimental procedures

### Cloning, expression, and purification

The full-length N-terminally His_6_-tagged constructs for cloning wildtype *csa3*_*Sso*_ (accession number: Sso1445) and *csa3*_*Sso*_ (R98A) mutant were synthesized from Twist Bio, Inc, and cloned into the *NdeI* and *EcoRI* sites of a pBB75 vector using In-Fusion Cloning (Takara Bio USA) generating plasmids pBB75(His_6_-Csa3_Sso__WT) and pBB75(His_6_-Csa3_Sso__R98A), respectively. For the generation of other *csa3*_*Sso*_ mutants targeting the Csa3_Sso_•cA4 structural interface (F10A, F14A, G96A, E122A and E122Q), the His_6_-tagged *csa3*_*Sso*_ (WT) was first cloned into *NdeI* and *EcoRI* sites of pET21b plasmid by PCR amplification from pBB75(His_6_-Csa3_Sso__WT) using primers pET21(N-His_6_-Csa3_sso__WT)_F and pET21(N-His_6_-Csa3_sso__WT)_R (Table S2). The plasmid thus generated, pET21(His_6_-Csa3_WT), was then subjected to Q5 site-directed mutagenesis (New England Biolabs) using manufacturer’s protocol and custom mutagenesis primers (identified with the prefix pET21 in Table S2).

For protein expression, the recombinant His_6_-tagged plasmids (pBB75 [His_6_-Csa3] and pET21b [His_6_-Csa3]) were transformed into BL21(DE3)-pLysS *E. coli* cells and selected on LB plates containing 0.03 mg/ml Kanamycin (for recombinant pBB75 plasmids) or 0.1 mg/ml Ampicillin (for recombinant pET21b plasmids). For the expression of the native and mutant Csa3_Sso_ proteins, a single *E. coli* transformant colony was grown in ZYP-5025 autoinduction media (89) containing 0.03 mg/ml Kanamycin (for recombinant pBB75 plasmids) or 0.1 mg/ml Ampicillin (for recombinant pET21b plasmids) at 22 °C for 44 to 48 h. Cells were harvested and resuspended in buffer A (20 mM Tris-HCl pH 8.0 and 400 mM NaCl) containing 0.1 mg/ml DNase I (Sigma-Aldrich), 5 mM β-mercaptoethanol, and 1% v/v protease inhibitor cocktail (Sigma-Aldrich). Cells were lysed using Emulsiflex-C3 (Avestin, Inc), and the lysates of His_6_-Csa3 were preheated for 30 min at 65 °C, respectively, and centrifuged at 14,000 rpm for 40 min to remove the cell debris.

The supernatants containing all the wildtype and mutant His_6_-Csa3_Sso_ preparations were applied to a HisTrap Fast Flow column (Cytiva Life Sciences, Inc) pre-equilibrated in buffer A. Following a wash with five column volumes (CVs) of buffer A, Csa3_Sso_ was eluted with a three-step gradient of buffer B (20 mM Tris-HCl pH 8.0 and 50 mM NaCl) to buffer C (20 mM Tris-HCl pH 8.0, 50 mM NaCl, and 500 mM Imidazole): (i) 0% to 10% (v/v) buffer C in ten CVs, (ii) 10% to 40% (v/v) buffer C in ten CVs, and (iii) 40% to 100% (v/v) buffer C in 20 CVs. The fractions containing Csa3_Sso_ were pooled and applied to a Mono-Q anion exchange chromatography column (Cytiva Life Sciences, Inc) equilibrated with buffer D (50 mM Tris-HCl pH 9.0). The flow-through containing Csa3_Sso_ was collected and subjected to a Cibacron Blue 3GA column (Sigma Aldrich) equilibrated with buffer D. Bound proteins were eluted using a linear gradient of buffer D and buffer E (50 mM Tris-HCl pH 9.0 and 1.5 M NaCl).

His_6_-tagged Csa3_Sso_ preparations obtained above were concentrated and applied onto a Superdex 200 16/70 gel filtration column pre-equilibrated with buffer F (20 mM Tris-HCl pH 8.0 and 50 mM NaCl). The Csa3_Sso_-containing fractions were concentrated using an Amicon Ultra-10 kDa cutoff centrifugal filter (Millipore) and stored at −80 °C.

### Microscale thermophoresis

The ligand-binding specificity of Csa3_Sso_ (WT) and the role of Csa3_Sso_ residues in ligand binding were determined by microscale Thermophoresis (MST). Wildtype or mutant His_6_-Csa3_Sso_ (200 nM) was fluorescently labeled in 2× binding buffer (10 mM Na/K phosphate, pH 5.8, 10 mM MgCl_2_, 25 mM NaCl, and 0.05% Tween 20) using 100 nM RED-Tris NTA His-Tag labeling dye (Nanotemper Technologies, Inc) and incubated in the dark at room temperature for 30 min. Two millimolar stocks of synthetic ligands including cA3, cA4, and cA6 (Biolog Life Science Institute) or linear ribonucleotide 5′-rCrArArArA-3′ (Bio-Synthesis Inc) were serially diluted in a 1:1 ratio with nuclease-free water. The labeled proteins were then mixed with ligands in a 1:1 ratio. Only cA4 was used in the MST experiments with the Csa3_Sso_ mutants (2 mM stocks of cA4 were used for F10A, F14A, G96A, and R98A mutants, and 20 μM stocks were used for E122A and E122Q mutants). The mixtures of proteins and ligands were incubated in the dark at room temperature for 30 min. The precipitated material was removed by centrifugation at 6000 rpm for 5 min. The supernatants were then loaded into Monolith NT.115 Series Premium capillaries in triplicate, and the thermophoresis was detected with 40% excitation power and 40% IR-laser power for an on-time of 20 s at 25 °C.

The MST experiment and data analysis for ligand-induced changes in Csa3_Sso_ binding affinity with P_Cas4a_ was performed as mentioned above except for the following: 1 μM of His_6_-Csa3_Sso_ (WT) was fluorescently labeled in 2× binding buffer with 40 nM RED-Tris NTA His-Tag labeling dye in the absence or presence of cA4 or cA6. The labeling mixtures were incubated in the dark at room temperature for 30 min. A 50 μM stock of P_Cas4a_ was serially diluted in 1:1 ratios with nuclease-free water. The labeled proteins ± ligands were then mixed with diluted P_Cas4a_ in a 1:1 ratio and incubated in the dark at room temperature for 30 min. The supernatants obtained after removal of precipitated material were then loaded into Monolith NT.115 Series Premium capillaries in triplicate, and the thermophoresis was detected with 100% excitation power and 60% IR-laser power for an on-time of 20 s at 25 °C.

The binding affinities of oligonucleotides and P_Cas4a_ to Csa3_Sso_ (WT and mutants) were analyzed according to the law of mass action in a standard fitting mode of MO.Affinity analysis software (version 2.3).

### Crystallization, X-ray data collection, data processing, model building, and refinement

Our high-throughput crystallization screen using His_6_-Csa3_Sso_ in the presence of 2-fold molar excess of cA4 identified a crystallization condition yielding cA4-dependent His_6_-Csa3_Sso_ crystals. The crystallization condition was optimized to obtain crystals growing up to 400 μm size. Csa3_Sso_•cA4 complex crystals were produced by the vapor diffusion method at 20 °C using a 1∶2 mixture of Csa3_Sso_•cA4 (300 μM His_6_-Csa3_Sso_ and 600 μM cA4) in gel filtration buffer (buffer F: 20 mM Tris-HCl pH 8.0 and 50 mM NaCl) and well solution (0.1 M K_2_SO_4_, 0.1 M Na/K 5.8, 16% PEG3350). X-ray diffraction data were collected in NSLS-II using AMX beamline at wavelength 0.92009 Å. The diffraction data were indexed, integrated, and scaled in HKL2000 (90). The initial phase information was obtained by molecular replacement in phenix phaser (91) using the native Csa3 structure (PDB ID: 2WTE) as template. The crystal belongs to P2_1_2_1_2 and two molecules of Csa3 are in an asymmetric unit. A strong difference density was observed on inspection of the F_O_-F_C_ map at the CARF domain dimeric interface and was identified as bound cA4. The molecular model for the ligand cA4 (CHEBI:142457) was obtained from the ChEBI EMBL database (92). The ligand restraints were generated in Phenix ReadySet, and cA4 has been manually modeled on the difference density map. Iterative rounds of manual model building in Coot and refinement in Phenix refinement generated the final model with R_work_ = 16.0 and R_free_ = 20.5 (93, 94). The stereochemical quality of the final structure was verified using Ramachandran plot, and 99.03% of the residues are found to have favorable conformation, whereas only 0.97% of residues have allowed conformation; no outlier was found. The structure is submitted to PDB with a PDB ID 6WXQ. The data processing and refinement statistics are reported in Table 1.

### Ring nuclease activity assays

The ring nuclease activity assays of cA4 were performed in two different conditions. In the first condition (used for Fig. S5, *A* and *B*), 50 μM Csa3_Sso_ and/or 250 μM cA4 was incubated (in a cA4:Csa3_Sso_ molar ratio of 5:1) at 55 °C for 3 h either in (i) 1× binding buffer or (ii) the buffer G (20 mM Tris-HCl, pH 7.5, 50 mM KCl, and 50 mM NaCl) previously used to demonstrate the ring nuclease activity of Csm6 (84). The reaction mixtures were deproteinized by ultrafiltration with an Amicon Ultra 3 kDa cutoff centrifugal filter (Millipore). In the second condition (used for Figs. 6 and S5*C*), 5 μM Csa3_Sso_ (wildtype or its E122A/E122Q mutants) and/or 500 μM cA_4_ was incubated (in a cA4:Csa3_Sso_ molar ratio of 100:1) in the buffer G or 1× binding buffer at 60 °C for 3 h. The reactions were quenched and deproteinized by phenol–chloroform extractions (84). For both conditions, the products and controls were collected and analyzed with High Performance Liquid Chromatography system (UltiMate 3000, Thermo Scientific) equipped with the C18 column (4.6 × 100 mm, 5 μM particle size, Thermo Scientific) and C18 column, (15 cm × 4.6 mm, 3 μM particle size, Supelco), for the first and second conditions, respectively.

### Analytical ultracentrifugation

Sedimentation velocity analytical ultracentrifugation (SV-AUC) experiments were performed at 20 °C with an XL-A analytical ultracentrifuge (Beckman-Coulter) and a TiAn60 rotor with two-channel charcoal-filled Epon centerpieces and quartz windows. Data were collected with detection at 280 nm. Complete sedimentation velocity profiles were recorded every 30 s at 40,000 rpm. Data were fit using the *c(S)* implementation of the Lamm equation as implemented in the program SEDFIT (95) and corrected for S_20,w_. Direct fitting of association models was performed using SEDPHAT (96). Calculated hydrodynamic properties for homology models were determined using WinHYDROPRO (97). The partial specific volume (ῡ), solvent density (ρ), and viscosity (η) were derived from chemical composition by SEDNTERP (http://www.rasmb.bbri.org/). Figures were created using the program GUSSI (98). All measurements were performed in 10 mM Na/KPO_4_ (pH 5.8), 10 mM NaCl, 25 mM MgCl_2_.

### Small-angle X-ray scattering data collection

SAXS data were collected at beamline 16-ID (LiX) of the National Synchrotron Light Source II (99). Data were collected at a wavelength of 1.0 Å in a three-camera conformation, yielding accessible scattering angle with 0.013 < q < 3.0 Å^−1^, where q is the momentum transfer, defined as q = 4π sin(θ)/λ, where λ is the X-ray wavelength and 2θ is the scattering angle. The data to q < 0.5 Å^−1^ were used in subsequent analyses. Samples were loaded into a 1-mm capillary for ten 1-s X-ray exposures. All measurements were performed in 10 mM Na/KPO_4_ (pH 5.8), 10 mM NaCl, 25 mM MgCl_2_.

### SAXS analysis

Data were analyzed in the program RAW (100). When fitting manually, the maximum diameter of the particle (D_max_) was incrementally adjusted in GNOM (101) to maximize the goodness-of-fit parameter, to minimize the discrepancy between the fit and the experimental data, and to optimize the visual qualities of the distribution profile.

Hybrid bead-atomistic modeling of Csa3 was performed using the program CORAL (70), where the known structure was fixed in composition and inventory not resolved by X-ray crystallography was modeled as coarse-grain beads. Ten independent calculations for each protein were performed and yielded comparable results. The final models were assessed using the program CRYSOL. The models were rendered using the program PYMOL (102).

### Electromobility shift assays

Top and bottom DNA oligonucleotide strands used for EMSA were purchased from Sigma-Aldrich (Table S2). The oligonucleotide strands were annealed by mixing them in 1:1 molar ratio followed by heating at 98 °C for the unlabeled, or 70 °C for the Cy5-labeled, DNA fragments for 15 min. Slow cooling to room temperature was used to anneal the fragments. Unlabeled probe-containing samples used for Fig. S8*C* were electrophoresed using a 2% (w/v) agarose Tris-Borate-EDTA (TBE) gel at 100 V for 30 min at room temperature. Probes were stained with ethidium bromide and visualized using Gel Doc XR+ Molecular Imager (Bio-Rad). EMSAs shown in Figure 9*C* were performed using the Cy5-labeled P_Cas4a_ probe. A 5′ amino-modified P_Cas4a_ top strand was obtained from Biosearch technologies. The Cy5-coupled oligo fraction was purified from unlabeled oligos and loose dye in C18 reverse-phase HPLC using already established protocols (103, 104). The Cy5-labeled probes were electrophoresed in a 5% acrylamide TBE gel at 100 V for 1 h at 4 °C. DNA bands were visualized using FluorChem R gel imager (Protein Simple, Inc).

## Data availability

Coordinates and structure factors for the Csa3_Sso_:cA4 structure have been deposited in the RCSB Protein Data Bank (http://www.rcsb.org) with the accession code 6WXQ. Strains and plasmids are described in this article, and the raw data for the binding analyses in Figures 1*D*, 4*E* and 9*D* and Fig. S1 are available upon request.

## Supporting information

This article contains supporting information (59, 61, 101, 102).

## Conflict of interest

The authors declare that they have no conflicts of interest with the contents of this article.

## References

[1] Barrangou R., Fremaux C., Deveau H., Richards M., Boyaval P., Moineau S., Romero D.A., Horvath P. (2007). CRISPR provides acquired resistance against viruses in prokaryotes. Science.

[2] Grissa I., Vergnaud G., Pourcel C. (2007). CRISPRFinder: A web tool to identify clustered regularly interspaced short palindromic repeats. Nucleic Acids Res..

[3] van der Oost J., Westra E.R., Jackson R.N., Wiedenheft B. (2014). Unravelling the structural and mechanistic basis of CRISPR-Cas systems. Nat. Rev. Microbiol..

[4] Yu Z., Jiang S., Wang Y., Tian X., Zhao P., Xu J., Feng M., She Q. (2021). CRISPR-Cas adaptive immune systems in Sulfolobales: Genetic studies and molecular mechanisms. Sci. China Life Sci..

[5] Ishino Y., Shinagawa H., Makino K., Amemura M., Nakata A. (1987). Nucleotide sequence of the iap gene, responsible for alkaline phosphatase isozyme conversion in Escherichia coli, and identification of the gene product. J. Bacteriol..

[6] Mojica F.J., Diez-Villasenor C., Garcia-Martinez J., Soria E. (2005). Intervening sequences of regularly spaced prokaryotic repeats derive from foreign genetic elements. J. Mol. Evol..

[7] Jansen R., Embden J.D., Gaastra W., Schouls L.M. (2002). Identification of genes that are associated with DNA repeats in prokaryotes. Mol. Microbiol..

[8] Hille F., Charpentier E. (2016). CRISPR-Cas: Biology, mechanisms and relevance. Philos. Trans. R. Soc. Lond. B Biol. Sci..

[9] Nunez J.K., Kranzusch P.J., Noeske J., Wright A.V., Davies C.W., Doudna J.A. (2014). Cas1-Cas2 complex formation mediates spacer acquisition during CRISPR-Cas adaptive immunity. Nat. Struct. Mol. Biol..

[10] Yosef I., Goren M.G., Qimron U. (2012). Proteins and DNA elements essential for the CRISPR adaptation process in *Escherichia coli*. Nucleic Acids Res..

[11] Lee H., Zhou Y., Taylor D.W., Sashital D.G. (2018). Cas4-dependent prespacer processing ensures high-fidelity programming of CRISPR arrays. Mol. Cell.

[12] Bhaya D., Davison M., Barrangou R. (2011). CRISPR-Cas systems in bacteria and archaea: Versatile small RNAs for adaptive defense and regulation. Annu. Rev. Genet..

[13] Brouns S.J., Jore M.M., Lundgren M., Westra E.R., Slijkhuis R.J., Snijders A.P., Dickman M.J., Makarova K.S., Koonin E.V., van der Oost J. (2008). Small CRISPR RNAs guide antiviral defense in prokaryotes. Science.

[14] Carte J., Wang R., Li H., Terns R.M., Terns M.P. (2008). Cas6 is an endoribonuclease that generates guide RNAs for invader defense in prokaryotes. Genes Dev..

[15] Hale C.R., Zhao P., Olson S., Duff M.O., Graveley B.R., Wells L., Terns R.M., Terns M.P. (2009). RNA-guided RNA cleavage by a CRISPR RNA-Cas protein complex. Cell.

[16] Lintner N.G., Kerou M., Brumfield S.K., Graham S., Liu H., Naismith J.H., Sdano M., Peng N., She Q., Copie V., Young M.J., White M.F., Lawrence C.M. (2011). Structural and functional characterization of an archaeal clustered regularly interspaced short palindromic repeat (CRISPR)-associated complex for antiviral defense (CASCADE). J. Biol. Chem..

[17] Zhang J., Rouillon C., Kerou M., Reeks J., Brugger K., Graham S., Reimann J., Cannone G., Liu H., Albers S.V., Naismith J.H., Spagnolo L., White M.F. (2012). Structure and mechanism of the CMR complex for CRISPR-mediated antiviral immunity. Mol. Cell.

[18] Reeks J., Naismith J.H., White M.F. (2013). CRISPR interference: A structural perspective. Biochem. J..

[19] Peng W., Li H., Hallstrom S., Peng N., Liang Y.X., She Q. (2013). Genetic determinants of PAM-dependent DNA targeting and pre-crRNA processing in *Sulfolobus islandicus*. RNA Biol..

[20] Westra E.R., Semenova E., Datsenko K.A., Jackson R.N., Wiedenheft B., Severinov K., Brouns S.J. (2013). Type I-E CRISPR-cas systems discriminate target from non-target DNA through base pairing-independent PAM recognition. PLoS Genet..

[21] Anders C., Niewoehner O., Duerst A., Jinek M. (2014). Structural basis of PAM-dependent target DNA recognition by the Cas9 endonuclease. Nature.

[22] Li M., Wang R., Xiang H. (2014). *Haloarcula hispanica* CRISPR authenticates PAM of a target sequence to prime discriminative adaptation. Nucleic Acids Res..

[23] Wang J., Li J., Zhao H., Sheng G., Wang M., Yin M., Wang Y. (2015). Structural and mechanistic basis of PAM-dependent spacer acquisition in CRISPR-Cas systems. Cell.

[24] Yamano T., Zetsche B., Ishitani R., Zhang F., Nishimasu H., Nureki O. (2017). Structural basis for the canonical and non-canonical PAM recognition by CRISPR-Cpf1. Mol. Cell.

[25] Makarova K.S., Wolf Y.I., Alkhnbashi O.S., Costa F., Shah S.A., Saunders S.J., Barrangou R., Brouns S.J., Charpentier E., Haft D.H., Horvath P., Moineau S., Mojica F.J., Terns R.M., Terns M.P. (2015). An updated evolutionary classification of CRISPR-Cas systems. Nat. Rev. Microbiol..

[26] Mohanraju P., Makarova K.S., Zetsche B., Zhang F., Koonin E.V., van der Oost J. (2016). Diverse evolutionary roots and mechanistic variations of the CRISPR-Cas systems. Science.

[27] Cong L., Ran F.A., Cox D., Lin S., Barretto R., Habib N., Hsu P.D., Wu X., Jiang W., Marraffini L.A., Zhang F. (2013). Multiplex genome engineering using CRISPR/Cas systems. Science.

[28] Doudna J.A., Charpentier E. (2014). Genome editing. The new frontier of genome engineering with CRISPR-Cas9. Science.

[29] Burstein D., Harrington L.B., Strutt S.C., Probst A.J., Anantharaman K., Thomas B.C., Doudna J.A., Banfield J.F. (2017). New CRISPR-Cas systems from uncultivated microbes. Nature.

[30] Makarova K.S., Haft D.H., Barrangou R., Brouns S.J., Charpentier E., Horvath P., Moineau S., Mojica F.J., Wolf Y.I., Yakunin A.F., van der Oost J., Koonin E.V. (2011). Evolution and classification of the CRISPR-Cas systems. Nat. Rev. Microbiol..

[31] Majumdar S., Zhao P., Pfister N.T., Compton M., Olson S., Glover C.V., Wells L., Graveley B.R., Terns R.M., Terns M.P. (2015). Three CRISPR-Cas immune effector complexes coexist in *Pyrococcus furiosus*. RNA.

[32] Elmore J.R., Sheppard N.F., Ramia N., Deighan T., Li H., Terns R.M., Terns M.P. (2016). Bipartite recognition of target RNAs activates DNA cleavage by the type III-B CRISPR-Cas system. Genes Dev..

[33] Zink I.A., Wimmer E., Schleper C. (2020). Heavily armed ancestors: CRISPR immunity and applications in archaea with a comparative analysis of CRISPR types in Sulfolobales. Biomolecules.

[34] Deng L., Garrett R.A., Shah S.A., Peng X., She Q. (2013). A novel interference mechanism by a type IIIB CRISPR-Cmr module in Sulfolobus. Mol. Microbiol..

[35] Manica A., Schleper C. (2013). CRISPR-mediated defense mechanisms in the hyperthermophilic archaeal genus Sulfolobus. RNA Biol..

[36] Zhang J., White M.F. (2013). Hot and crispy: CRISPR-Cas systems in the hyperthermophile *Sulfolobus solfataricus*. Biochem. Soc. Trans..

[37] Hille F., Richter H., Wong S.P., Bratovic M., Ressel S., Charpentier E. (2018). The biology of CRISPR-Cas: Backward and forward. Cell.

[38] Mulepati S., Heroux A., Bailey S. (2014). Structural biology. Crystal structure of a CRISPR RNA-guided surveillance complex bound to a ssDNA target. Science.

[39] Hayes R.P., Xiao Y., Ding F., van Erp P.B., Rajashankar K., Bailey S., Wiedenheft B., Ke A. (2016). Structural basis for promiscuous PAM recognition in type I-E cascade from E. coli. Nature.

[40] Li Y., Zhang Y., Lin J., Pan S., Han W., Peng N., Liang Y.X., She Q. (2017). Cmr1 enables efficient RNA and DNA interference of a III-B CRISPR-Cas system by binding to target RNA and crRNA. Nucleic Acids Res..

[41] Pan S., Li Q., Deng L., Jiang S., Jin X., Peng N., Liang Y., She Q., Li Y. (2019). A seed motif for target RNA capture enables efficient immune defence by a type III-B CRISPR-Cas system. RNA Biol..

[42] Goldberg G.W., Jiang W., Bikard D., Marraffini L.A. (2014). Conditional tolerance of temperate phages via transcription-dependent CRISPR-Cas targeting. Nature.

[43] Samai P., Pyenson N., Jiang W., Goldberg G.W., Hatoum-Aslan A., Marraffini L.A. (2015). Co-transcriptional DNA and RNA cleavage during type III CRISPR-Cas immunity. Cell.

[44] Estrella M.A., Kuo F.T., Bailey S. (2016). RNA-activated DNA cleavage by the type III-B CRISPR-Cas effector complex. Genes Dev..

[45] Kazlauskiene M., Tamulaitis G., Kostiuk G., Venclovas C., Siksnys V. (2016). Spatiotemporal control of type III-A CRISPR-Cas immunity: Coupling DNA degradation with the target RNA recognition. Mol. Cell.

[46] Han W., Li Y., Deng L., Feng M., Peng W., Hallstrom S., Zhang J., Peng N., Liang Y.X., White M.F., She Q. (2017). A type III-B CRISPR-Cas effector complex mediating massive target DNA destruction. Nucleic Acids Res..

[47] Liu T.Y., Iavarone A.T., Doudna J.A. (2017). RNA and DNA targeting by a reconstituted Thermus thermophilus type III-A CRISPR-Cas system. PLoS One.

[48] Silas S., Lucas-Elio P., Jackson S.A., Aroca-Crevillen A., Hansen L.L., Fineran P.C., Fire A.Z., Sanchez-Amat A. (2017). Type III CRISPR-Cas systems can provide redundancy to counteract viral escape from type I systems. Elife.

[49] Pyenson N.C., Gayvert K., Varble A., Elemento O., Marraffini L.A. (2017). Broad targeting specificity during bacterial type III CRISPR-Cas immunity constrains viral escape. Cell Host Microbe.

[50] Rouillon C., Athukoralage J.S., Graham S., Gruschow S., White M.F. (2018). Control of cyclic oligoadenylate synthesis in a type III CRISPR system. Elife.

[51] Kazlauskiene M., Kostiuk G., Venclovas C., Tamulaitis G., Siksnys V. (2017). A cyclic oligonucleotide signaling pathway in type III CRISPR-Cas systems. Science.

[52] Niewoehner O., Garcia-Doval C., Rostol J.T., Berk C., Schwede F., Bigler L., Hall J., Marraffini L.A., Jinek M. (2017). Type III CRISPR-Cas systems produce cyclic oligoadenylate second messengers. Nature.

[53] Koonin E.V., Makarova K.S. (2018). Discovery of oligonucleotide signaling mediated by CRISPR-associated polymerases solves two puzzles but leaves an enigma. ACS Chem. Biol..

[54] Athukoralage J.S., White M.F. (2021). Cyclic oligoadenylate signalling and regulation by ring nucleases during type III CRISPR defence. RNA.

[55] Quax T.E., Voet M., Sismeiro O., Dillies M.A., Jagla B., Coppee J.Y., Sezonov G., Forterre P., van der Oost J., Lavigne R., Prangishvili D. (2013). Massive activation of archaeal defense genes during viral infection. J. Virol..

[56] Haft D.H., Selengut J., Mongodin E.F., Nelson K.E. (2005). A guild of 45 CRISPR-associated (Cas) protein families and multiple CRISPR/Cas subtypes exist in prokaryotic genomes. PLoS Comput. Biol..

[57] Ye Q., Zhao X., Liu J., Zeng Z., Zhang Z., Liu T., Li Y., Han W., Peng N. (2020). CRISPR-associated factor Csa3b regulates CRISPR adaptation and Cmr-mediated RNA interference in Sulfolobus islandicus. Front. Microbiol..

[58] He F., Vestergaard G., Peng W., She Q., Peng X. (2017). CRISPR-Cas type I-A cascade complex couples viral infection surveillance to host transcriptional regulation in the dependence of Csa3b. Nucleic Acids Res..

[59] Liu T., Liu Z., Ye Q., Pan S., Wang X., Li Y., Peng W., Liang Y., She Q., Peng N. (2017). Coupling transcriptional activation of CRISPR-Cas system and DNA repair genes by Csa3a in Sulfolobus islandicus. Nucleic Acids Res..

[60] Liu T., Li Y., Wang X., Ye Q., Li H., Liang Y., She Q., Peng N. (2015). Transcriptional regulator-mediated activation of adaptation genes triggers CRISPR de novo spacer acquisition. Nucleic Acids Res..

[61] Lintner N.G., Frankel K.A., Tsutakawa S.E., Alsbury D.L., Copie V., Young M.J., Tainer J.A., Lawrence C.M. (2011). The structure of the CRISPR-associated protein Csa3 provides insight into the regulation of the CRISPR/Cas system. J. Mol. Biol..

[62] Lowey B., Whiteley A.T., Keszei A.F.A., Morehouse B.R., Mathews I.T., Antine S.P., Cabrera V.J., Kashin D., Niemann P., Jain M., Schwede F., Mekalanos J.J., Shao S., Lee A.S.Y., Kranzusch P.J. (2020). CBASS immunity uses CARF-related effectors to sense 3'-5'- and 2'-5'-linked cyclic oligonucleotide signals and protect bacteria from phage infection. Cell.

[63] Rostol J.T., Xie W., Kuryavyi V., Maguin P., Kao K., Froom R., Patel D.J., Marraffini L.A. (2021). The Card1 nuclease provides defence during type III CRISPR immunity. Nature.

[64] Athukoralage J.S., Graham S., Rouillon C., Gruschow S., Czekster C.M., White M.F. (2020). The dynamic interplay of host and viral enzymes in type III CRISPR-mediated cyclic nucleotide signalling. Elife.

[65] Krissinel E. (2010). Crystal contacts as nature's docking solutions. J. Comput. Chem..

[66] Krissinel E., Henrick K. (2007). Inference of macromolecular assemblies from crystalline state. J. Mol. Biol..

[67] Makarova K.S., Timinskas A., Wolf Y.I., Gussow A.B., Siksnys V., Venclovas C., Koonin E.V. (2020). Evolutionary and functional classification of the CARF domain superfamily, key sensors in prokaryotic antivirus defense. Nucleic Acids Res..

[68] Athukoralage J.S., Rouillon C., Graham S., Gruschow S., White M.F. (2018). Ring nucleases deactivate type III CRISPR ribonucleases by degrading cyclic oligoadenylate. Nature.

[69] McMahon S.A., Zhu W., Graham S., Rambo R., White M.F., Gloster T.M. (2020). Structure and mechanism of a type III CRISPR defence DNA nuclease activated by cyclic oligoadenylate. Nat. Commun..

[70] Petoukhov M.V., Franke D., Shkumatov A.V., Tria G., Kikhney A.G., Gajda M., Gorba C., Mertens H.D., Konarev P.V., Svergun D.I. (2012). New developments in the ATSAS program package for small-angle scattering data analysis. J. Appl. Crystallogr..

[71] Hong M., Fuangthong M., Helmann J.D., Brennan R.G. (2005). Structure of an OhrR-ohrA operator complex reveals the DNA binding mechanism of the MarR family. Mol. Cell.

[72] Hidalgo-Cantabrana C., Barrangou R. (2020). Characterization and applications of type I CRISPR-Cas systems. Biochem. Soc. Trans..

[73] Semenova E., Jore M.M., Datsenko K.A., Semenova A., Westra E.R., Wanner B., van der Oost J., Brouns S.J., Severinov K. (2011). Interference by clustered regularly interspaced short palindromic repeat (CRISPR) RNA is governed by a seed sequence. Proc. Natl. Acad. Sci. U. S. A..

[74] Deveau H., Barrangou R., Garneau J.E., Labonte J., Fremaux C., Boyaval P., Romero D.A., Horvath P., Moineau S. (2008). Phage response to CRISPR-encoded resistance in Streptococcus thermophilus. J. Bacteriol..

[75] Marino N.D., Zhang J.Y., Borges A.L., Sousa A.A., Leon L.M., Rauch B.J., Walton R.T., Berry J.D., Joung J.K., Kleinstiver B.P., Bondy-Denomy J. (2018). Discovery of widespread type I and type V CRISPR-Cas inhibitors. Science.

[76] Gussow A.B., Park A.E., Borges A.L., Shmakov S.A., Makarova K.S., Wolf Y.I., Bondy-Denomy J., Koonin E.V. (2020). Machine-learning approach expands the repertoire of anti-CRISPR protein families. Nat. Commun..

[77] Niewoehner O., Jinek M. (2017). Specialized weaponry: How a type III-A CRISPR-Cas system excels at combating phages. Cell Host Microbe.

[78] Watson B.N.J., Steens J.A., Staals R.H.J., Westra E.R., van Houte S. (2021). Coevolution between bacterial CRISPR-Cas systems and their bacteriophages. Cell Host Microbe.

[79] Mo C.Y., Mathai J., Rostol J.T., Varble A., Banh D.V., Marraffini L.A. (2021). Type III-A CRISPR immunity promotes mutagenesis of staphylococci. Nature.

[80] Hanukoglu I. (2015). Proteopedia: Rossmann fold: A beta-alpha-beta fold at dinucleotide binding sites. Biochem. Mol. Biol. Educ..

[81] Makarova K.S., Anantharaman V., Grishin N.V., Koonin E.V., Aravind L. (2014). CARF and WYL domains: Ligand-binding regulators of prokaryotic defense systems. Front. Genet..

[82] Aasland R., Abrams C., Ampe C., Ball L.J., Bedford M.T., Cesareni G., Gimona M., Hurley J.H., Jarchau T., Lehto V.P., Lemmon M.A., Linding R., Mayer B.J., Nagai M., Sudol M. (2002). Normalization of nomenclature for peptide motifs as ligands of modular protein domains. FEBS Lett..

[83] Molina R., Stella S., Feng M., Sofos N., Jauniskis V., Pozdnyakova I., Lopez-Mendez B., She Q., Montoya G. (2019). Structure of Csx1-cOA4 complex reveals the basis of RNA decay in type III-B CRISPR-Cas. Nat. Commun..

[84] Jia N., Jones R., Yang G., Ouerfelli O., Patel D.J. (2019). CRISPR-Cas III-A Csm6 CARF domain is a ring nuclease triggering stepwise cA4 cleavage with ApA>p formation terminating RNase activity. Mol. Cell.

[85] Athukoralage J.S., Graham S., Gruschow S., Rouillon C., White M.F. (2019). A type III CRISPR ancillary ribonuclease degrades its cyclic oligoadenylate activator. J. Mol. Biol..

[86] Athukoralage J.S., McQuarrie S., Gruschow S., Graham S., Gloster T.M., White M.F. (2020). Tetramerisation of the CRISPR ring nuclease Crn3/Csx3 facilitates cyclic oligoadenylate cleavage. Elife.

[87] Athukoralage J.S., McMahon S.A., Zhang C., Gruschow S., Graham S., Krupovic M., Whitaker R.J., Gloster T.M., White M.F. (2020). An anti-CRISPR viral ring nuclease subverts type III CRISPR immunity. Nature.

[88] Garcia-Doval C., Schwede F., Berk C., Rostol J.T., Niewoehner O., Tejero O., Hall J., Marraffini L.A., Jinek M. (2020). Activation and self-inactivation mechanisms of the cyclic oligoadenylate-dependent CRISPR ribonuclease Csm6. Nat. Commun..

[89] Studier F.W. (2005). Protein production by auto-induction in high density shaking cultures. Protein Expr. Purif..

[90] Otwinowski Z., Minor W. (1997). Processing of X-ray diffraction data collected in oscillation mode. Methods Enzymol..

[91] McCoy A.J., Grosse-Kunstleve R.W., Adams P.D., Winn M.D., Storoni L.C., Read R.J. (2007). Phaser crystallographic software. J. Appl. Crystallogr..

[92] Hastings J., Owen G., Dekker A., Ennis M., Kale N., Muthukrishnan V., Turner S., Swainston N., Mendes P., Steinbeck C. (2016). ChEBI in 2016: Improved services and an expanding collection of metabolites. Nucleic Acids Res..

[93] Adams P.D., Afonine P.V., Bunkoczi G., Chen V.B., Davis I.W., Echols N., Headd J.J., Hung L.W., Kapral G.J., Grosse-Kunstleve R.W., McCoy A.J., Moriarty N.W., Oeffner R., Read R.J., Richardson D.C. (2010). PHENIX: A comprehensive Python-based system for macromolecular structure solution. Acta Crystallogr. D Biol. Crystallogr..

[94] Emsley P., Cowtan K. (2004). Coot: Model-building tools for molecular graphics. Acta Crystallogr. D Biol. Crystallogr..

[95] Schuck P. (2000). Size-distribution analysis of macromolecules by sedimentation velocity ultracentrifugation and lamm equation modeling. Biophys. J..

[96] Vistica J., Dam J., Balbo A., Yikilmaz E., Mariuzza R.A., Rouault T.A., Schuck P. (2004). Sedimentation equilibrium analysis of protein interactions with global implicit mass conservation constraints and systematic noise decomposition. Anal. Biochem..

[97] Ortega A., Amoros D., Garcia de la Torre J. (2011). Prediction of hydrodynamic and other solution properties of rigid proteins from atomic- and residue-level models. Biophys. J..

[98] Brautigam C.A. (2015). Calculations and publication-quality illustrations for analytical ultracentrifugation data. Methods Enzymol..

[99] DiFabio J., Chodankar S., Pjerov S., Jakoncic J., Lucas M., Krywka C., Graziano V., Yang L. (2017). The life science x-ray scattering beamline at NSLS-II. AIP Conf. Proc..

[100] Hopkins J.B., Gillilan R.E., Skou S. (2017). BioXTAS RAW: Improvements to a free open-source program for small-angle X-ray scattering data reduction and analysis. J. Appl. Crystallogr..

[101] Semenyuk A.V., Svergun D.I. (1991). Gnom - a program package for small-angle scattering data-processing. J. Appl. Crystallogr..

[102] DeLano W.L. (2004). Use of PYMOL as a communications tool for molecular science. Abstr. Pap. Am. Chem. S.

[103] Batish M., Raj A., Tyagi S. (2011). Single molecule imaging of RNA *in situ*. Methods Mol. Biol..

[104] Batish M., Tyagi S. (2019). Fluorescence in situ imaging of dendritic RNAs at single-molecule resolution. Curr. Protoc. Neurosci..

[105] Rambo R.P., Tainer J.A. (2011). Characterizing flexible and intrinsically unstructured biological macromolecules by SAS using the Porod-Debye law. Biopolymers.

[106] Rambo R.P., Tainer J.A. (2013). Accurate assessment of mass, models and resolution by small-angle scattering. Nature.

[107] Poirot O., O'Toole E., Notredame C. (2003). Tcoffee@igs: A web server for computing, evaluating and combining multiple sequence alignments. Nucleic Acids Res..

