# Supplementary material for: Structural basis of cyclic oligoadenylate binding to the transcription factor Csa3 outlines cross talk between type III and type I CRISPR systems
Source: J Biol Chem. 2022 Jan 14;298(2):101591. doi: 10.1016/j.jbc.2022.101591 (PMC8844856; doi:10.1016/j.jbc.2022.101591)
Supplement: Supplemental Figures S1–S9 and Tables S1, S2 [file mmc1.pdf]

SUPPORTING MATERIAL

Structural basis of cyclic oligoadenylate binding to the transcription factor Csa3 outlines crosstalk between Type-III & Type-I CRISPR systems

Pengjun Xia[2], Anirudha Dutta[1], Kushol Gupta[3], Mona Batish[1,2], and Vijay Parashar[1,2]*

[1]Department of Medical and Molecular Sciences, 15 Innovation Way, University of Delaware, Newark, DE 19711

[2]Department of Biological Sciences, University of Delaware, Newark, DE 19716

[3]The Department of Biochemistry and Biophysics, Perelman School of Medicine, University of Pennsylvania, Philadelphia, PA 19104, USA.

Supplementary Table 1. Parameters derived from SV-AUC c(S) analysis.

Sample	Loaded Concentration (µM)	Peak $S_{20,w}$	f/f_o	M_f (kDa)	RMSD
Experimental parameters					
Csa3$_{Sso}$	96	3.5	1.30	52.0	0.005
Csa3$_{Sso}$ + 50 µM cA4	40	3.6	1.39	61.2	0.005
Csa3$_{Sso}$ (R98A)	64	3.5	1.40	57.4	0.005
Calculated parameters from structural models					
Csa3$_{Sso}$ alone (PDB 2WTE)		3.8		52.3	
Csa3$_{Sso}$ bound with cA4 (This Study)		3.86		57.5	

Supplementary Table 2. Nucleotide sequences of oligonucleotides used in this study.

**Oligonucleotide name**		**Oligonucleotide sequence (5’ to 3’)**
**P_{Cas4a}**	Top strand	AAAGAATTCCCGAATTTACTAGGGAT
	Bottom strand	ATCCCTAGTAAATTCGGGAATTCTTT
**$P_{Sso\_LeaderA}$**	Top strand	AATCCTTGCGACCAGAAATTGTTAAA
	Bottom strand	TTTAACAATTTCTGGTCGCAAGGATT
**Negative control**	Top strand	CAATCTAGGACTTTATACCTAGT
	Bottom strand	ACTAGGTATAAAGTCCTAGATTG
**pET21(N-His$_6$-Csa3$_{sso}$_WT)_F**		GAAGGAGATATACATATGCATCACCATCACCATCAC
**pET21(N-His$_6$-Csa3$_{sso}$_WT)_R**		TCGACGGAGCTCGAATTCCTAGTAAGGGATGTTTAC
**pET21(N-His$_6$-Csa3$_{sso}$_F10A)_F**		TTTTTGTTGCGTCTTCTTAATGAG
**pET21(N-His$_6$-Csa3$_{sso}$_F10A)_R**		AGTCTCGTTCGCTCCCATTGTCAC
**pET21(N-His$_6$-Csa3$_{sso}$_F14A)_F**		CTTCTTAATGAGACATCTGCGC
**pET21(N-His$_6$-Csa3$_{sso}$_F14A)_R**		ACGCAACAACGCAGTCTCGTTG
**pET21(N-His$_6$-Csa3$_{sso}$_G96A)_F**		GATCTGACAATGGCGATGCGTATG
**pET21(N-His$_6$-Csa3$_{sso}$_G96A)_R**		CGAGATGATCGGCTCTGGCAGCG
**pET21(N-His$_6$-Csa3$_{sso}$_E122A)_F**		GTTCGTGACGCGGGTGGAGGAAG
**pET21(N-His$_6$-Csa3$_{sso}$_E122Q)_F**		GTTCGTGACCAGGGTGGAGGAAG
**N-His$_6$-Csa3$_{sso}$ (E122A)/(E122Q)_R**		GTAGACCGTGAAGCGCTTACGGG
**pBB75(N-His$_6$-Csa3$_{sso}$_WT)**		Gene block sequence:<br>CATCACCATCACCATCACCATGGTGCTGCAATGAAATCATA<br>TTTCGTGACAATGGGATTCAACGAGACTTTTTTGTTGCGTC<br>TTCTTAATGAGACATCTGCGCAGAAAGAAGATAGTCTGGT<br>CATCGTGGTCCCCTCCCCAATTGTCAGCGGGACCCGCGCT<br>GCTATCGAATCCCTTCGCGCGCAAATTAGCCGCTTAAATTA<br>TCCTCCACCCCGTATTTATGAAATTGAGATTACCGACTTCA<br>ACCTGGCACTTAGTAAAATCCTGGATATTATTCTGACGCTG<br>CCAGAGCCGATCATCTCGGATCTGACAATGGGGATGCGTA<br>TGATCAACACCTTGATTCTGCTTGGAATCATTGTCTCCCGT<br>AAGCGCTTCACGGTCTACGTTCGTGACGAGGGTGGAGGA<br>AGTCGCGTTATTTCTTTCAACGATAACACAATTCGTGCGTT<br>AATGCGTGACTACAGCCGTGAAGAAATGAAACTTTTAAAT<br>GTTCTTTATGAGACTAAAGGTACTGGGATTACCGAATTGG<br>CAAAGATGTTAGACAAGAGTGAGAAAACTCTTATCAACAA<br>AATTGCGGAACTTAAGAAATTTGGGATTCTTACGCAAAAG<br>GGAAAGGATCGTAAGGTTGAACTGAATGAGTTGGGGTTA<br>AATGTGATTAAATTGAATAAATCCGTGATCGAGAGTAGTA<br>AATCGAGTGAAGAGCTTGTGAAGGAGAATAAGGGCAAAG<br>AAGTAAACATCCCTTACTAG

Supplementary Table 2 continued

Oligonucleotide name	Oligonucleotide sequence (5’ to 3’)
**pBB75(N-His$_6$-Csa3$_{sso}$_R98A)**	Gene block sequence:<br>CATCACCATCACCATCACCATGGTGCTGCAATGAAATCATA<br>TTTCGTGACAATGGGATTCAACGAGACTTTTTTGTTGCGTC<br>TTCTTAATGAGACATCTGCGCAGAAAGAAGATAGTCTGGT<br>CATCGTGGTCCCCTCCCCAATTGTCAGCGGGACCCGCGCT<br>GCTATCGAATCCCTTCGCGCGCAAATTAGCCGCTTAAATTA<br>TCCTCCACCCCGTATTTATGAAATTGAGATTACCGACTTCA<br>ACCTGGCACTTAGTAAAATCCTGGATATTATTCTGACGCTG<br>CCAGAGCCGATCATCTCGGATCTGACAATGGGGATGGCG<br>ATGATCAACACCTTGATTCTGCTTGGAATCATTGTCTCCCG<br>TAAGCGCTTCACGGTCTACGTTCGTGACGAGGGTGGAGG<br>AAGTCGCGTTATTTCTTTCAACGATAACACAATTCGTGCGT<br>TAATGCGTGACTACAGCCGTGAAGAAATGAAACTTTTAAA<br>TGTTCTTTATGAGACTAAAGGTACTGGGATTACCGAATTG<br>GCAAAGATGTTAGACAAGAGTGAGAAAACTCTTATCAACA<br>AAATTGCGGAACTTAAGAAATTTGGGATTCTTACGCAAAA<br>GGGAAAGGATCGTAAGGTTGAACTGAATGAGTTGGGGTT<br>AAATGTGATTAAATTGAATAAATCCGTGATCGAGAGTAGT<br>AAATCGAGTGAAGAGCTTGTGAAGGAGAATAAGGGCAAA<br>GAAGTAAACATCCCTTACTAG

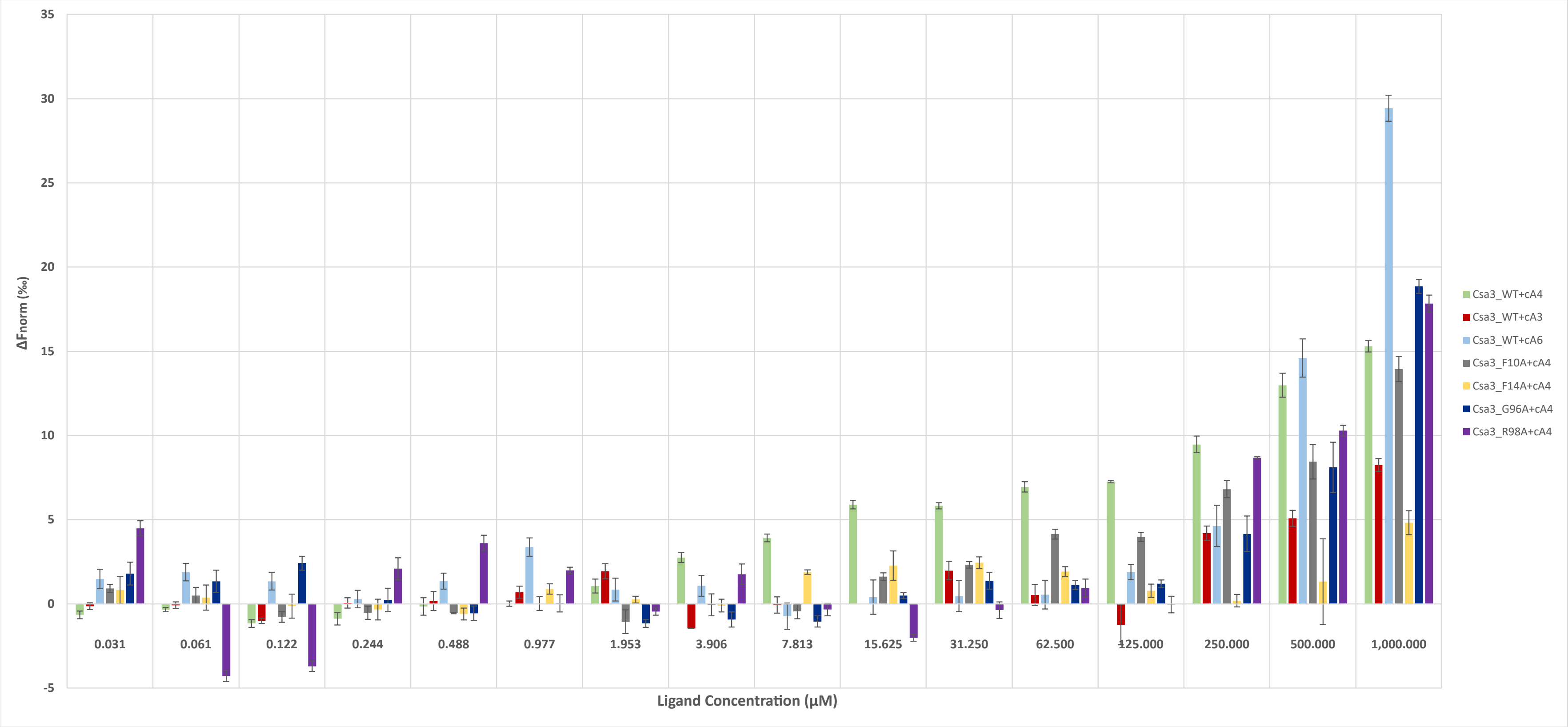

Figure S1. Non-specific cOA binding activity in Csa3$_{Sso}$. A non-specific cOA -dependent wild-type Csa3$_{Sso}$ (100nM) thermophoresis was detected for cA_3, cA_4 and cA_6 at high (≥ 250µM) cOA concentrations. This low affinity cA4 binding was also exhibited by the alanine mutants of Csa3$_{Sso}$ residues at structural interface with cA4, including F10, F14, G96 and R98. The graph displays data from three independent MST measurements. Error bars represent the standard deviation.

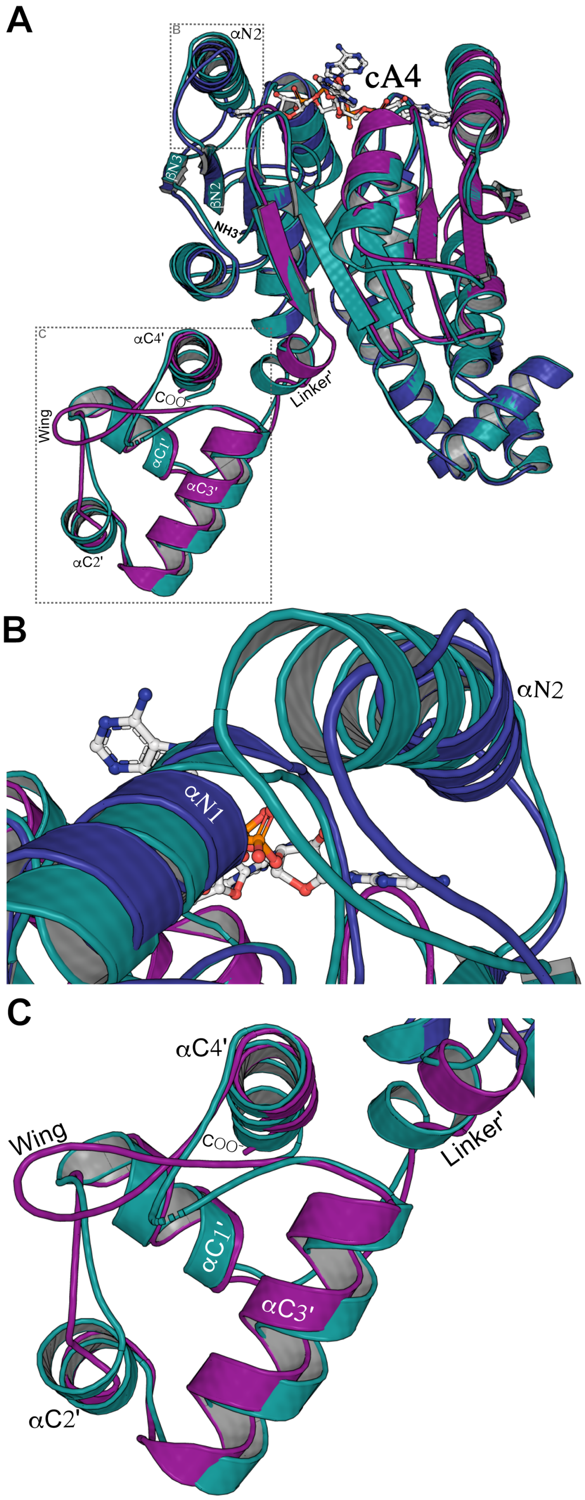

Figure S2. Comparison of Csa3$_{Sso}$•cA4 structure with apo Csa3$_{Sso}$. ***A,*** Superposition of the overall Csa3$_{Sso}$•cA4 structure (shown in a cartoon representation with blue and purple protomers) with apo Csa3$_{Sso}$ structure (PDB ID: 2WTE) (overall RMSD = 0.735 Å). ***B,*** Zoomed-in view of the protomer A CARF domain (outlined by a box in panel ***A***) showing change in the αN2 conformation. ***C,*** Zoomed-in view of the protomer B wHTH domain (outlined by a box in panel ***A***) showing stabilization of the 'wing' residues.

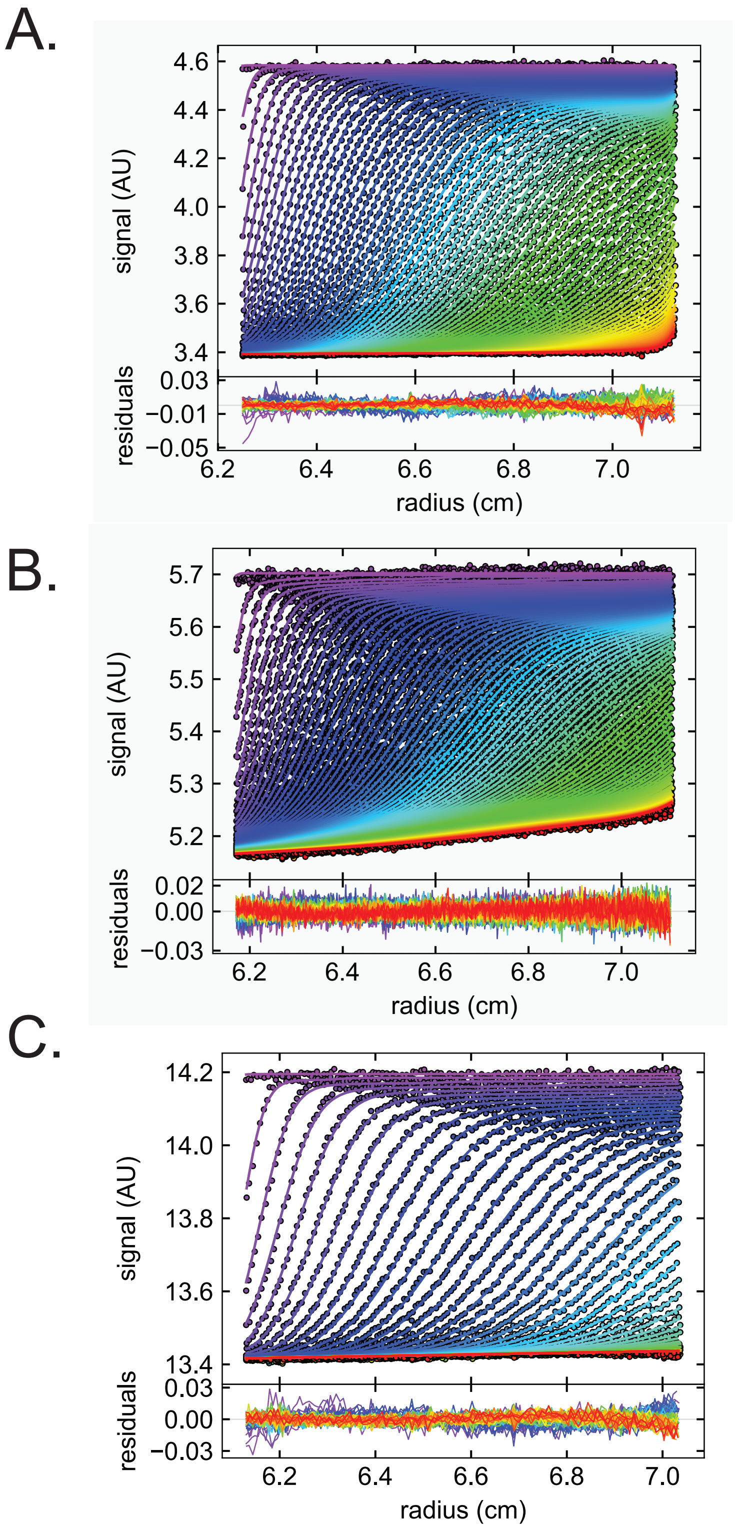

Figure S3 (related to Figure 3). Sedimentation velocity analytical ultracentrifugation (SV-AUC). Primary data for Csa3$_{Sso}$ dimers in the Apo (***A***) and cA4-bound (***B***) states of wild-type Csa3$_{Sso}$ as well as apo Csa3$_{Sso}$ -R98A mutant (***C***) are shown. In the uppermost panels for the samples shown, fits of the experimental data (circles) to the Lamm equation are shown as lines; in middle panel the residuals from this fitting are shown. Every third boundary and third datapoint are shown for clarity. Parameters derived from this fitting are detailed in Supplemental Table 1. Figures were prepared using the program GUSSI.

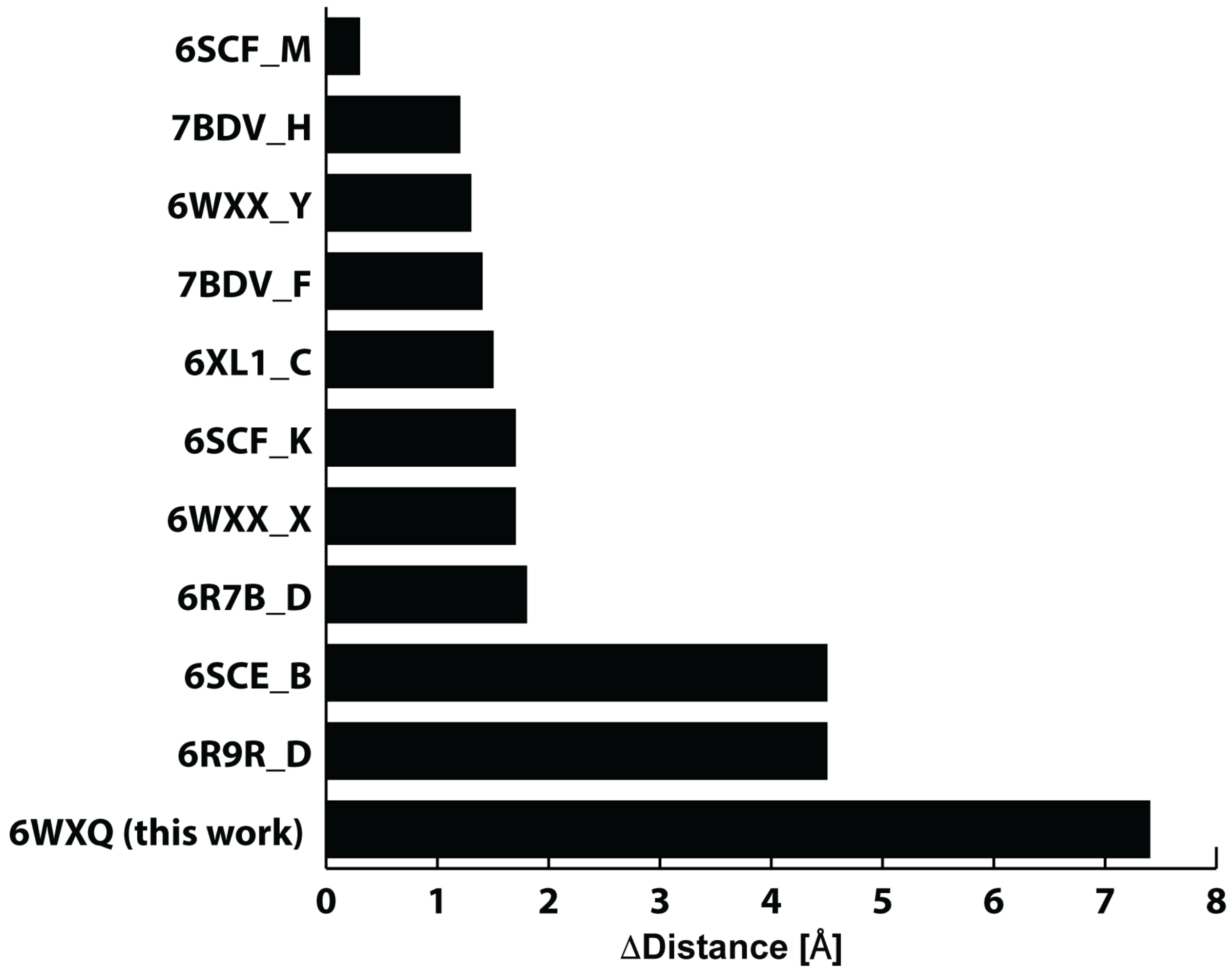

Figure S4. Csa3$_{Sso}$ -bound cA4 exists in an elongated conformation. The known cA4 conformations from different receptor-bound structures including the Csa3$_{Sso}$•cA4 structure from this work (PDB ID and chains depicted on the Y-axis) were analyzed and plotted for the difference between the distances (in Å) of distal phosphoryls from the proximal ones (labeled as ΔDistance on the X-axis).

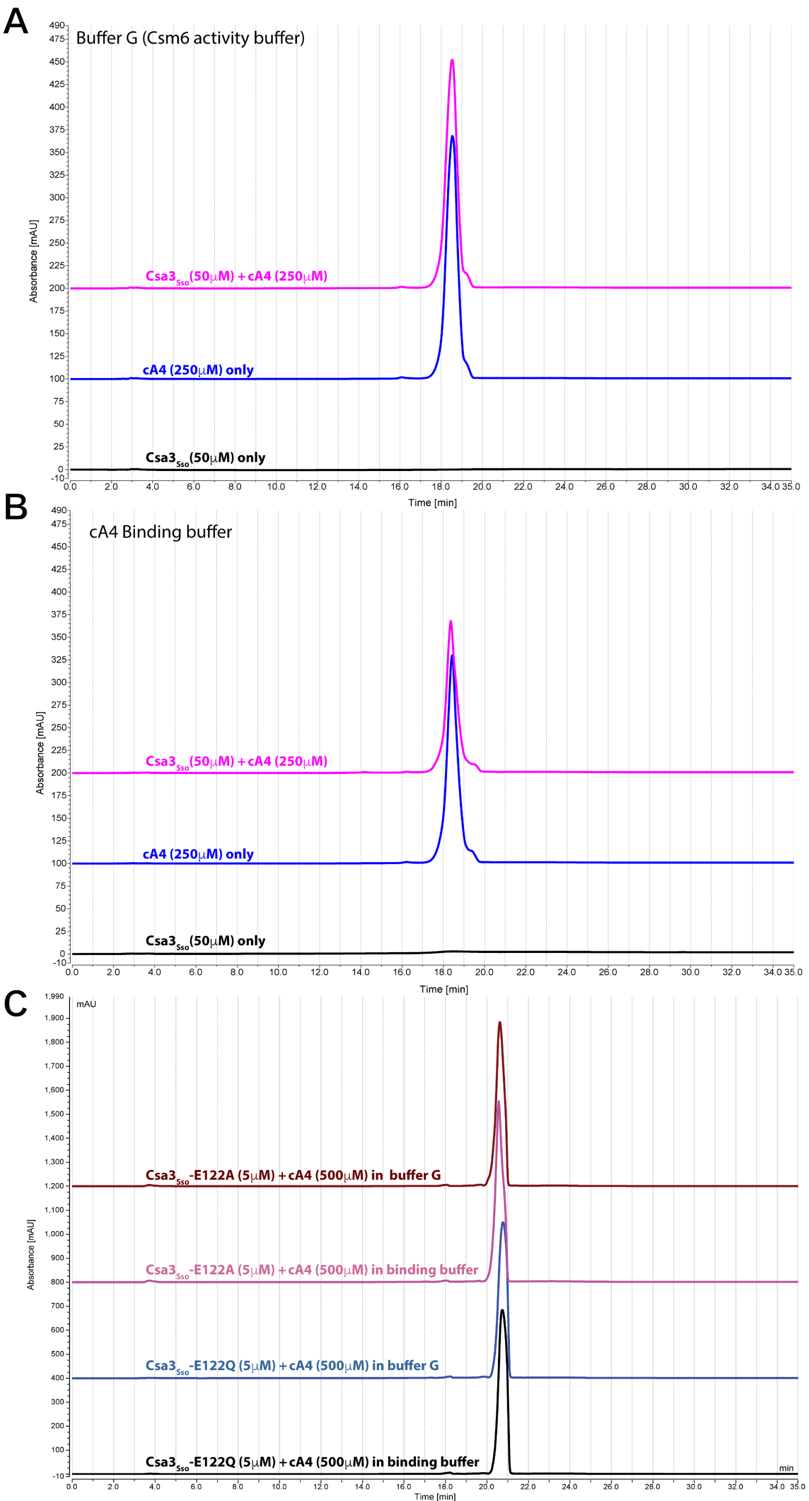

Figure S5. Wild-type and Glu122 mutants of Csa3$_{Sso}$ proteins show lack of cA4 ring nuclease activity. ***A & B,*** No changes in the time-dependent stability of cA4 were observed in reactions containing 50 µM wild-type Csa3$_{Sso}$ and 250 µM cA4 in buffer G (20 mM Tris-HCl, pH 7.5, 50 mM KCl and 50 mM NaCl) (panel ***A***)**,** or cA4 binding buffer (5 mM Na/K phosphate, pH 5.8, 5 mM $MgCl_2$, 12.5 mM NaCl, and 0.025 % Tween 20) (panel ***B***)**.** The reaction mixtures were deproteinized by ultrafiltration before C18-HPLC analysis. ***B,*** Csa3$_{Sso}$-E122A and Csa3$_{Sso}$-E122Q mutants also did not exhibit any gain of ring nuclease activity. The reactions were quenched and deproteinized by phenol-chloroform before C18-HPLC analysis.

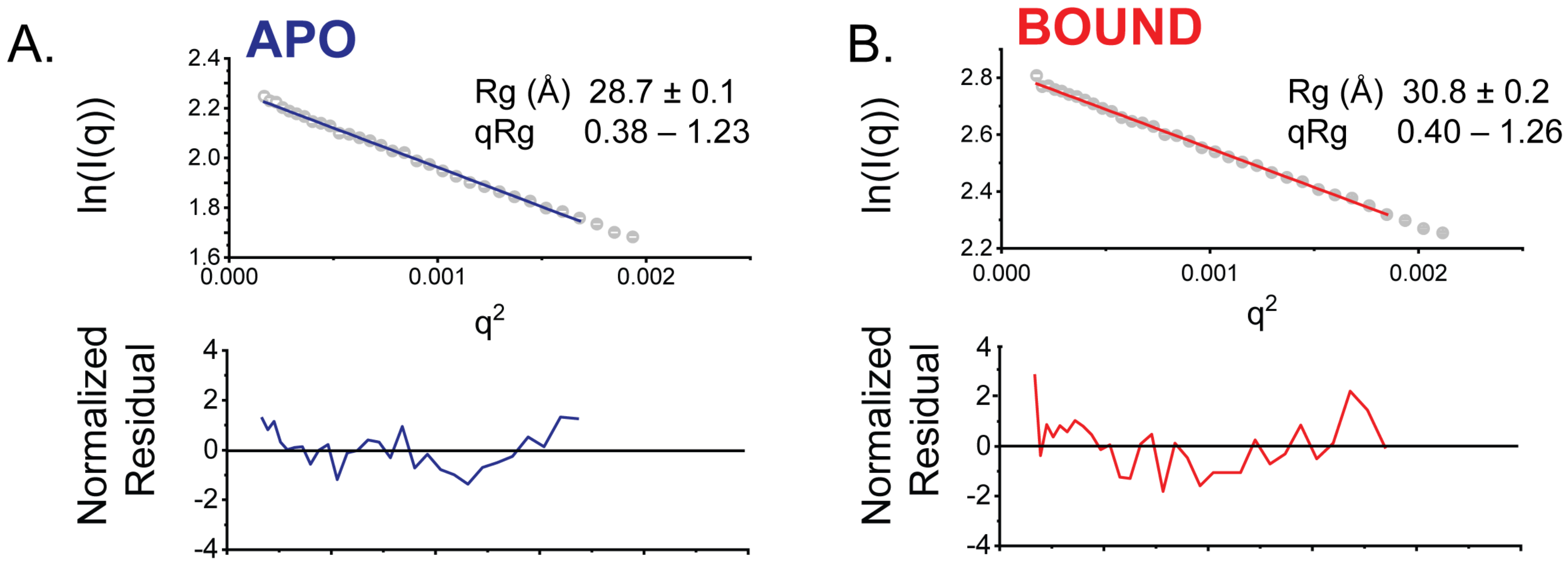

Figure S6 (related to Figure 7). Guinier Analysis. Shown are classical Guinier plots analyses (ln (I) vs. q^2) of SAXS data (circles) derived for Csa3$_{Sso}$ alone (A, blue) and in the presence of cA4 (B, red), with residuals from the fit lines shown below the respective fits. Plots were linear and indicative of profiles for monodisperse samples. Monodispersity is evidenced by linearity in the Guinier region of the scattering data and agreement of the I(0) and R_g values determined with inverse Fourier transform analysis by the programs GNOM (1). Guinier analyses were performed where $qR_g \leq 1.3$.

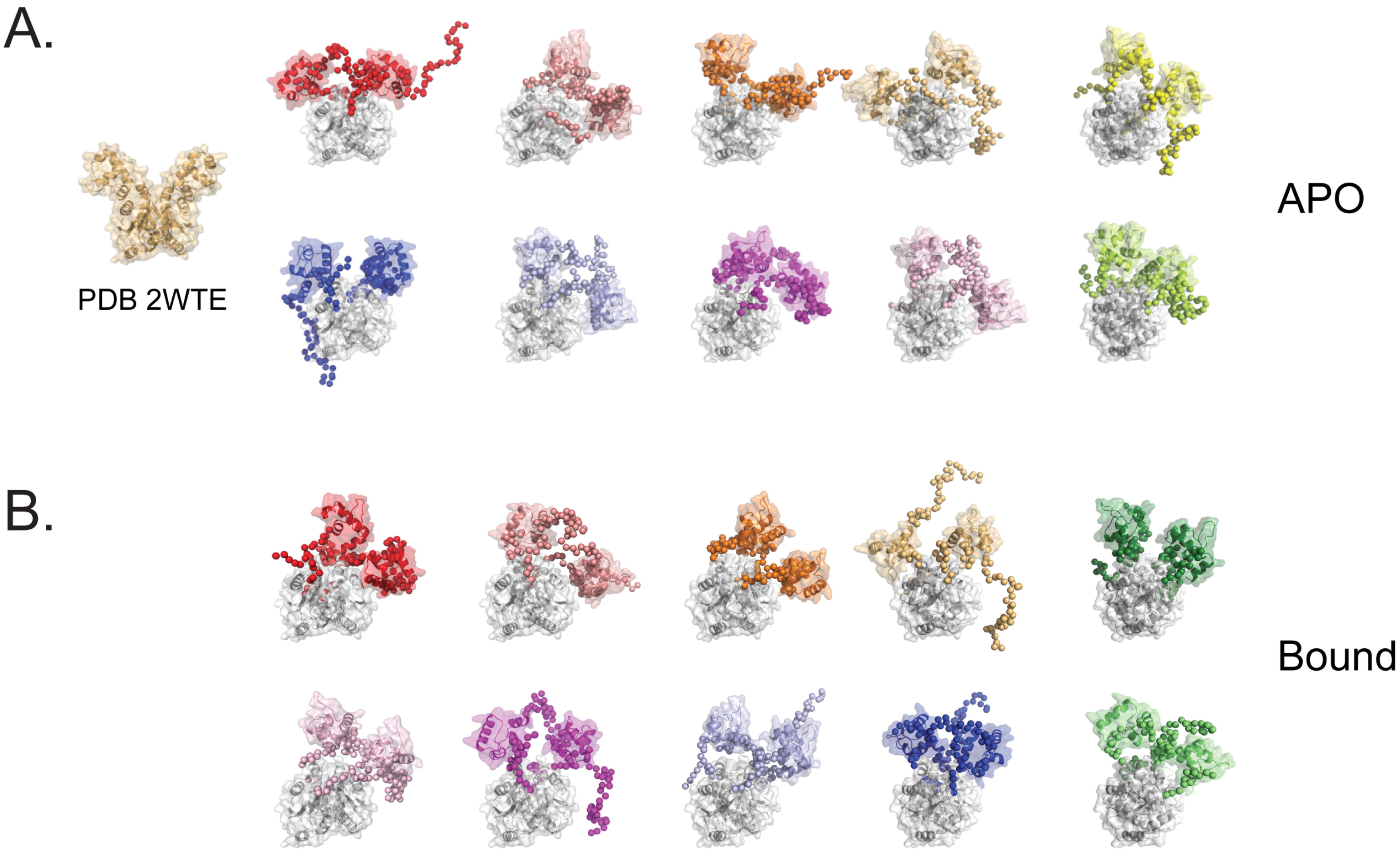

Figure S7 (related to Figure 7). Gallery of CORAL Results. A gallery of representative calculations (n=10) where the C-terminal wHTH domains were refined in atomic position. In the apo state (upper panel labeled Apo), χ^2_{Crysol} ranged from 1.5-2.1. In the cA4-bound state (lower panel labeled Bound), χ^2_{Crysol} ranged from 1.1-2.7. Atomic inventory not represented in available crystallographic models is shown as beads and was fit flexibly. The CARF domain dimer is shown in grey and the C-terminal wHTH domains are colored. Figures were rendered using PYMOL (2).

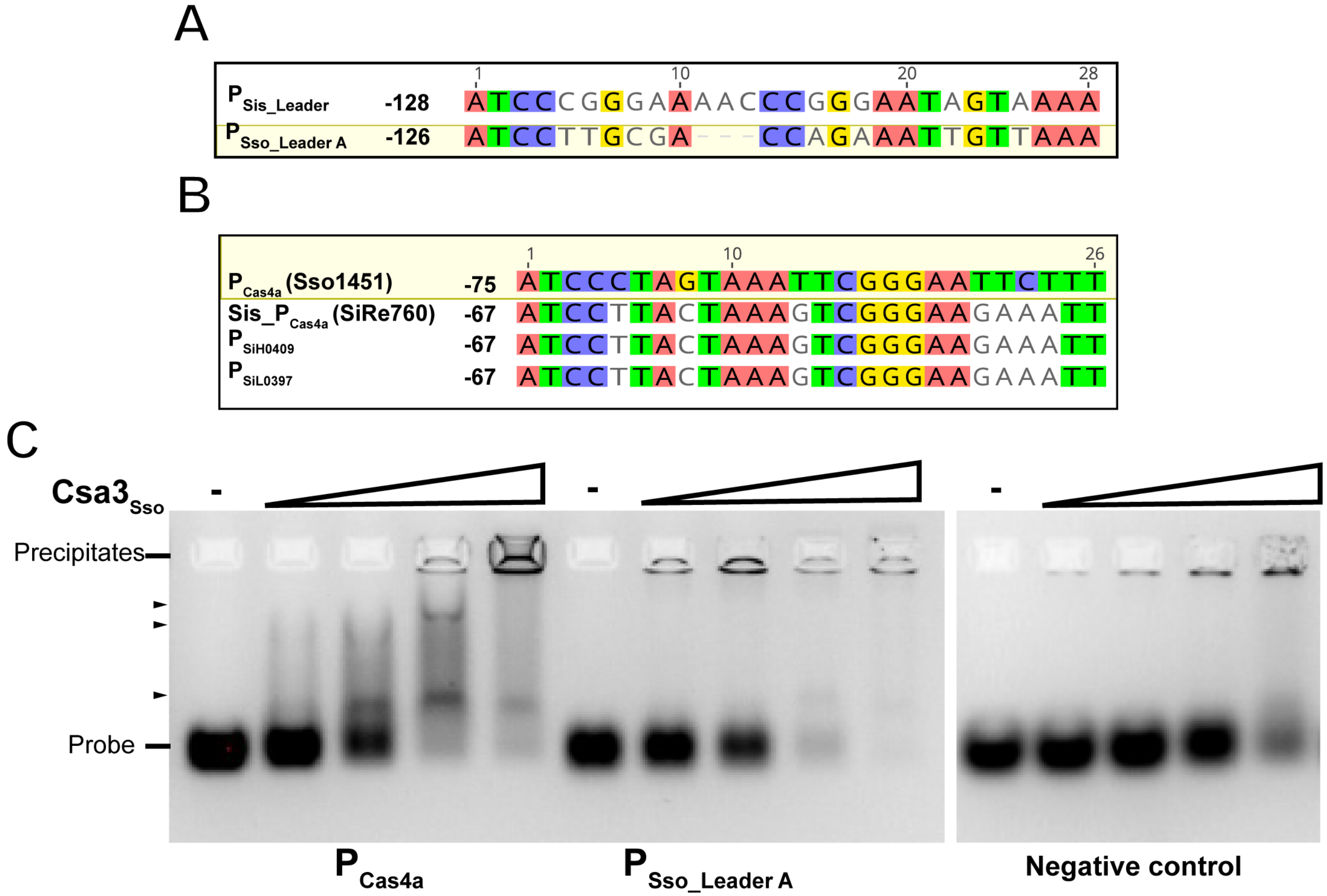

Figure S8. Preliminary identification of CRISPR promoter regions that show specific binding to Csa3$_{Sso}$. ***A&B)*** Sequence alignments of the previously predicted Csa3a$_{Sis}$ binding sites with *S. solfataricus* P2 CRISPR leader A and P$_{Cas4a}$ sequences (used in **panel C)**. Alignment of *S. solfataricus* P2 leader A (Sso_Leader A, highlighted in yellow background) with Sis Leader sequence (**panel A**), and *S. solfataricus* P2 cas4a promoter region (P$_{Cas4a}$, highlighted in yellow background) with various cas gene promoter regions previously predicted to bind Csa3a (3) (**panel B**). ***C)*** Identification of the P$_{Cas4a}$ fragment as a sequence-specific Csa3$_{Sso}$ binding site by agarose gel-based EMSAs. P$_{Cas4a}$ (10 µM), Sso_Leader A (5µM), or a CRISPR-unrelated (negative control) DNA (5µM) were mixed with Csa3$_{Sso}$ in the Csa3$_{Sso}$: DNA molar ratios of 1:1, 2:1, 5:1, and 10:1 for all the lanes labeled with triangles. While sequence-independent precipitation of Csa3$_{Sso}$-DNA complexes was seen for all the probes, only P$_{Cas4a}$ allowed formation of soluble Csa3$_{Sso}$-DNA complexes that entered the gel and moved as shifted bands (see triangles).

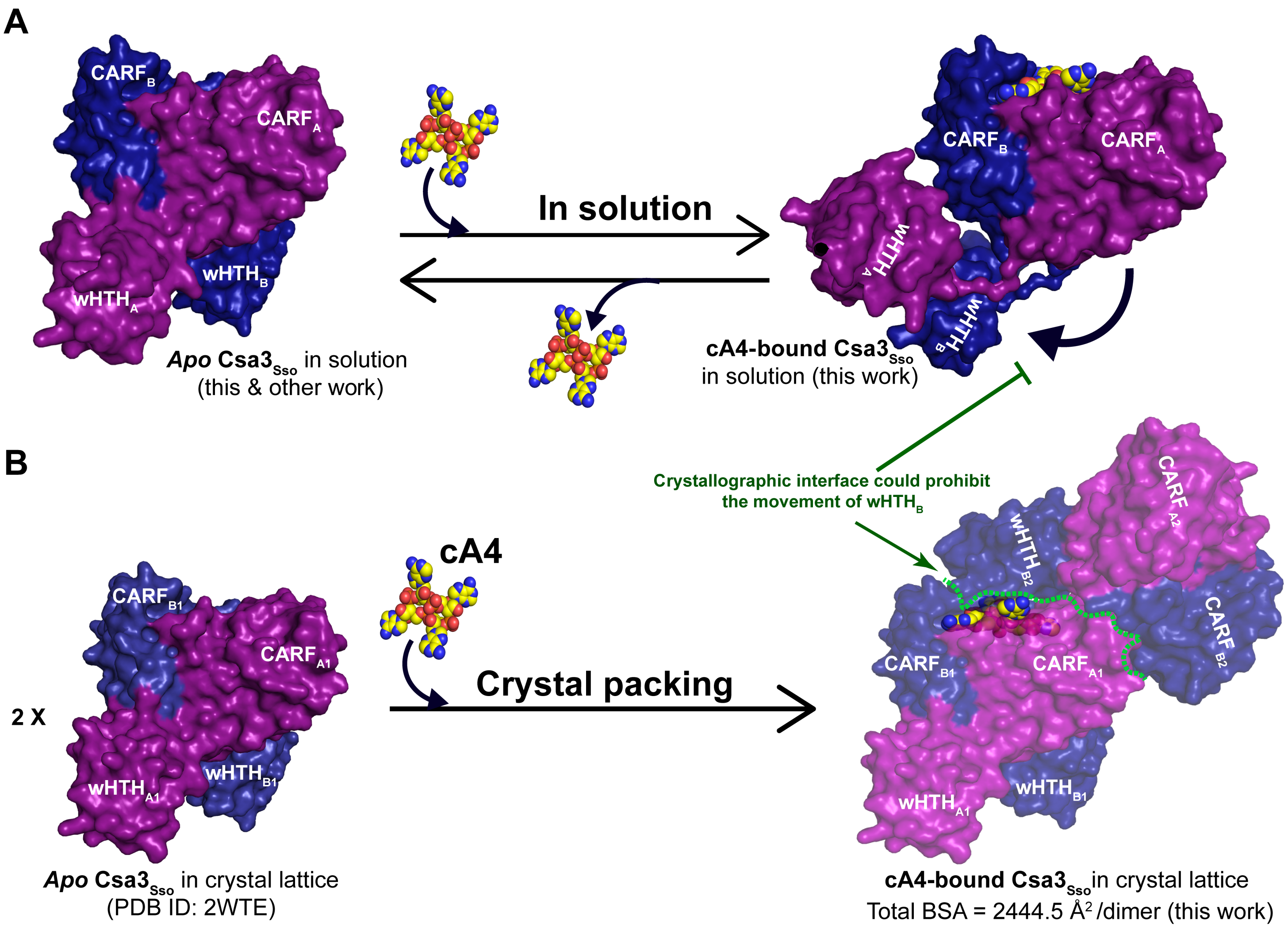

Figure S9. Comparison of the crystal and solution structures of Csa3$_{Sso}$. ***A)*** Conformational changes undertaken by wHTH$_A$ and wHTH$_B$ domains in the SAXS-derived Csa3 dimer in its cA4-bound state (this work) is compared to its *apo* state (this and previous work) (4). ***B)*** Crystal packing of the two cA4-bound Csa3$_{Sso}$ dimers (obtained by symmetry operator= X-1/2, -Y+1/2, -Z-1) is illustrated in surface representation. Extensive packing interactions between CARF$_{A+B}$ from the first Csa3$_{Sso}$ dimer copy, and wHTH$_B$ from the second Csa3$_{Sso}$ dimer copy (burying a significant surface area) could prohibit the in-solution movement of wHTH$_B$ (shown wHTH$_{B1}$).

References

1. Semenyuk, A. V., and Svergun, D. I. (1991) Gnom - a Program Package for Small-Angle Scattering Data-Processing. *Journal of Applied Crystallography* **24**, 537-540
2. DeLano, W. L. (2004) Use of PYMOL as a communications tool for molecular science. *Abstr Pap Am Chem S* **228**, U313-U314
3. Liu, T., Liu, Z., Ye, Q., Pan, S., Wang, X., Li, Y., Peng, W., Liang, Y., She, Q., and Peng, N. (2017) Coupling transcriptional activation of CRISPR-Cas system and DNA repair genes by Csa3a in Sulfolobus islandicus. *Nucleic Acids Res* **45**, 8978-8992
4. Lintner, N. G., Frankel, K. A., Tsutakawa, S. E., Alsbury, D. L., Copie, V., Young, M. J., Tainer, J. A., and Lawrence, C. M. (2011) The structure of the CRISPR-associated protein Csa3 provides insight into the regulation of the CRISPR/Cas system. *J Mol Biol* **405**, 939-955
